# Supplementary material for: Predicting Impacts of Climate Change on Northward Range Expansion of Invasive Weeds in South Korea
Source: Plants (Basel). 2021 Aug 5;10(8):1604. doi: 10.3390/plants10081604 (PMC8401637; doi:10.3390/plants10081604)
Supplement: Supplementary file 1 [file plants-10-01604-s001.zip › Supplementary figures S1 and S2.pdf]

**Figure S1a-p.** The Jackknife test results for indicating the relative contribution of environmental variable for modeling different invasive weeds. **S1**a, *Apium leptophyllum*; S1b, *Astragalus sinicus*; S1c, *Bromus unioloides*; S1d, *Chenopodium ambrosioides*; S1e, *Coronopus didymus*; S1f, *Gnaphalium calviceps*; S1g, *Lolium multiflorum* var. *multiflorum*; S1h, *Modiola caroliniana*; S1i, *Oenothera laciniate*; S1j, *Paspalum dilatatum*; S1k, *Sida rhombifolia*; S1l, *Silene gallica* var. *gallica*; S1m, *Sisymbrium officinale*; S1n, *Sisyrinchium angustifolium*; S1o, *Spergularia* rubra; S1p, *Malva parviflora*.

**Figure S2a-p.** The area under the receiver operating characteristics (ROC) curve for determing the model's goodness of fit under the current climate. **S2**a, *Apium leptophyllum*; S2b, *Astragalus sinicus*; S2c, *Bromus unioloides*; S2d, *Chenopodium ambrosioides*; S2e, *Coronopus didymus*; S2f, *Gnaphalium calviceps*; S2g, *Lolium multiflorum* var. *multiflorum*; S2h, *Modiola caroliniana*; S2i, *Oenothera laciniate*; S2j, *Paspalum dilatatum*; S2k, *Sida rhombifolia*; S2l, *Silene gallica* var. *gallica*; S2m, *Sisymbrium officinale*; S2n, *Sisyrinchium angustifolium*; S2o, *Spergularia* rubra; S2p, *Malva parviflora*.

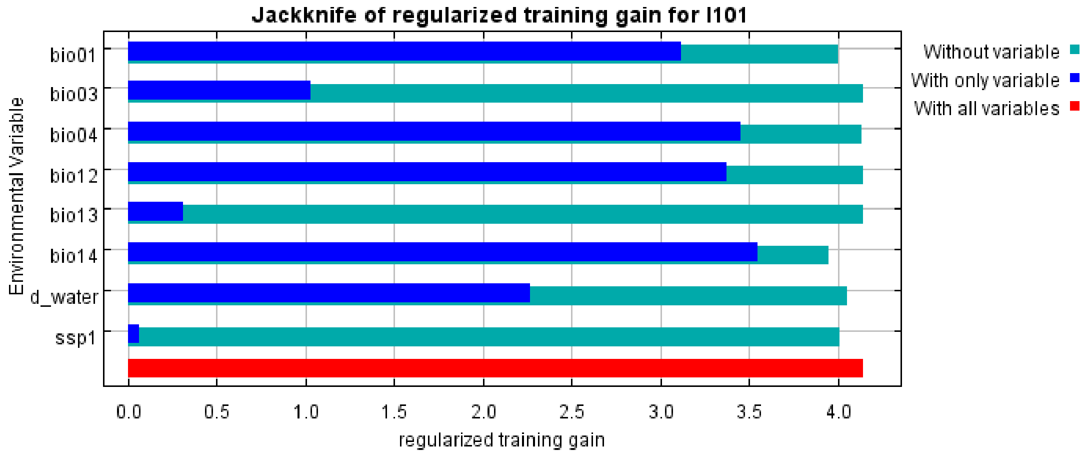


Figure S1a. The Jackknife test results for indicating the relative contribution of environmental varibales for *Apium leptophyllum*

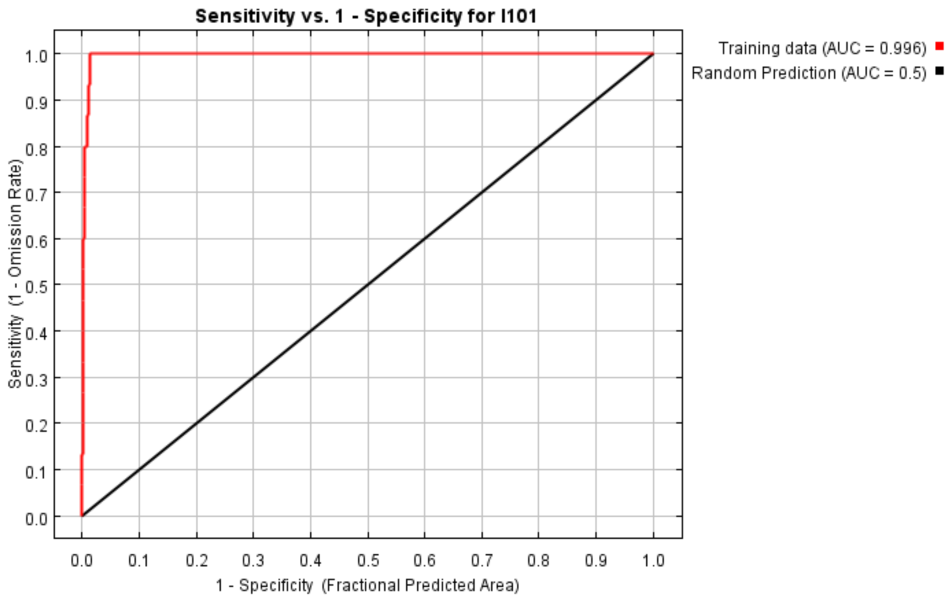


Figure S2a. The ROC curve for *Apium leptophyllum* under current climate.

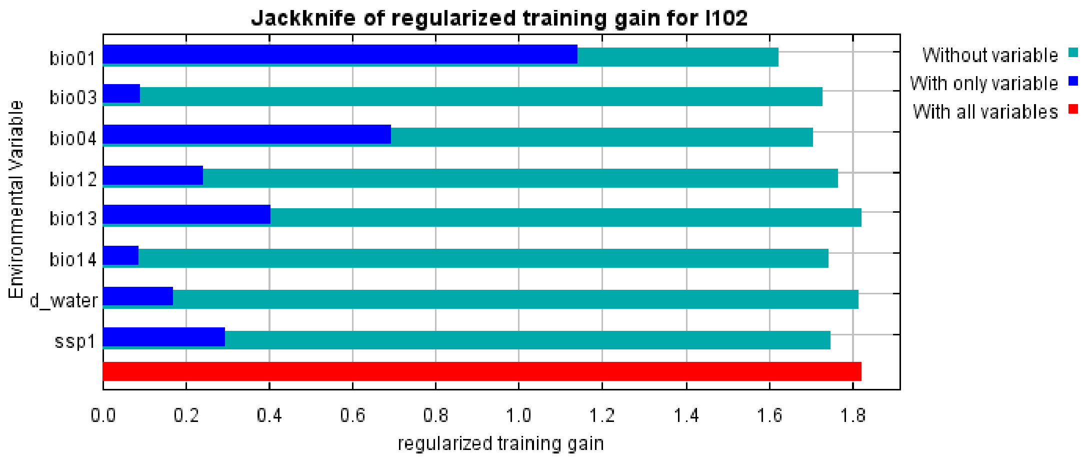


Figure S1b. The Jackknife test results for indicating the relative contribution of environmental varibales for *Astragalus sinicus*

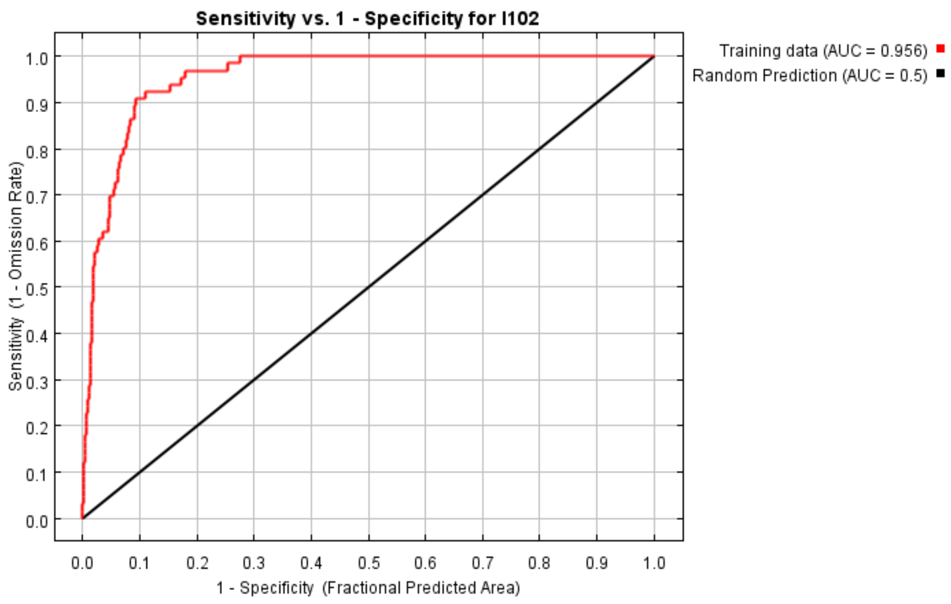


Figure S2b. The ROC curve for *Astragalus sinicus* under current climate.

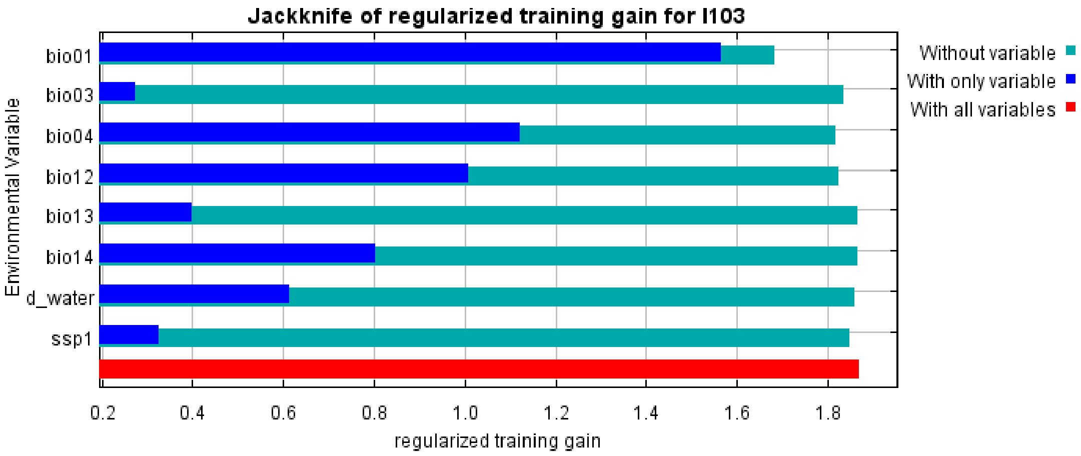


Figure S1c. The Jackknife test results for indicating the relative contribution of environmental varibales for *Bromus unioloides*

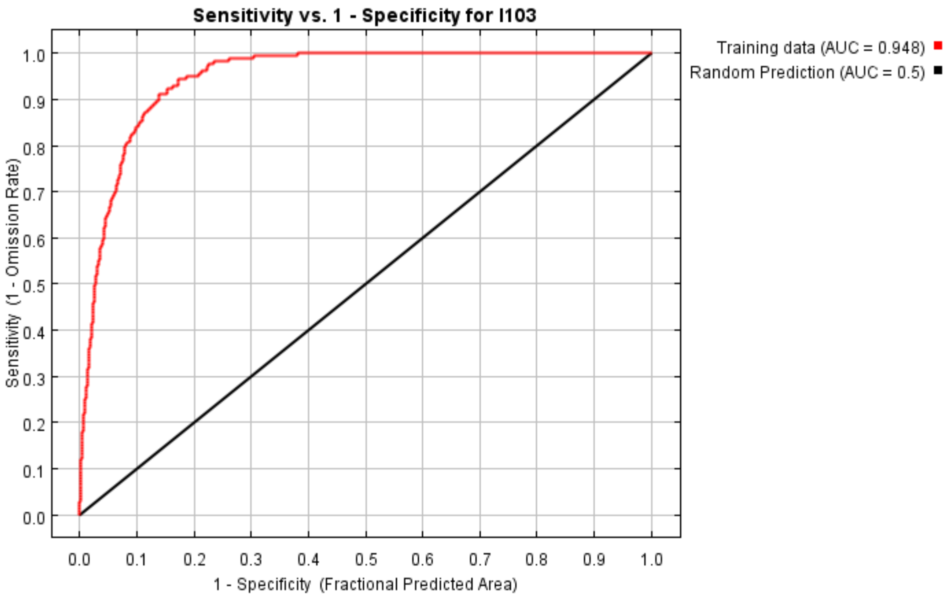


Figure S2c. The ROC curve for *Bromus unioloides* under current climate.

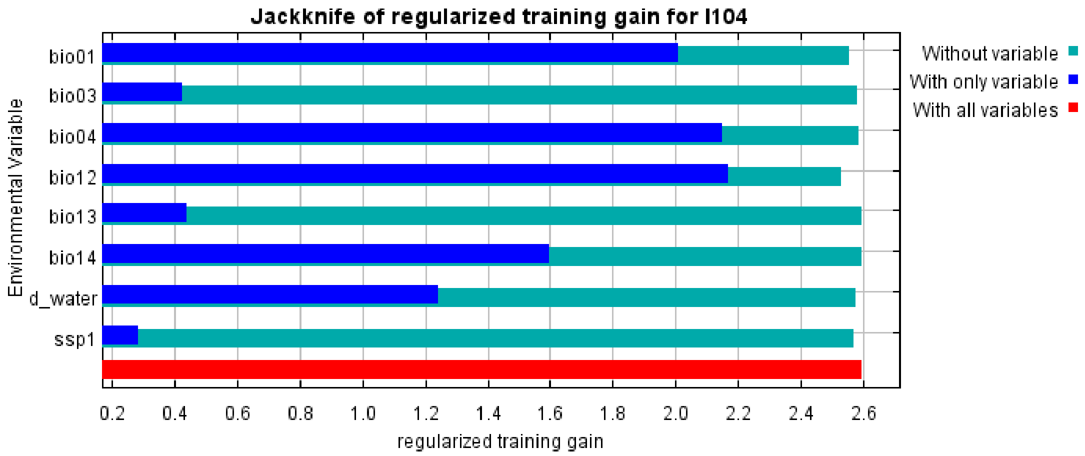


Figure S1d. The Jackknife test results for indicating the relative contribution of environmental varibales for *Chenopodium ambrosioides*

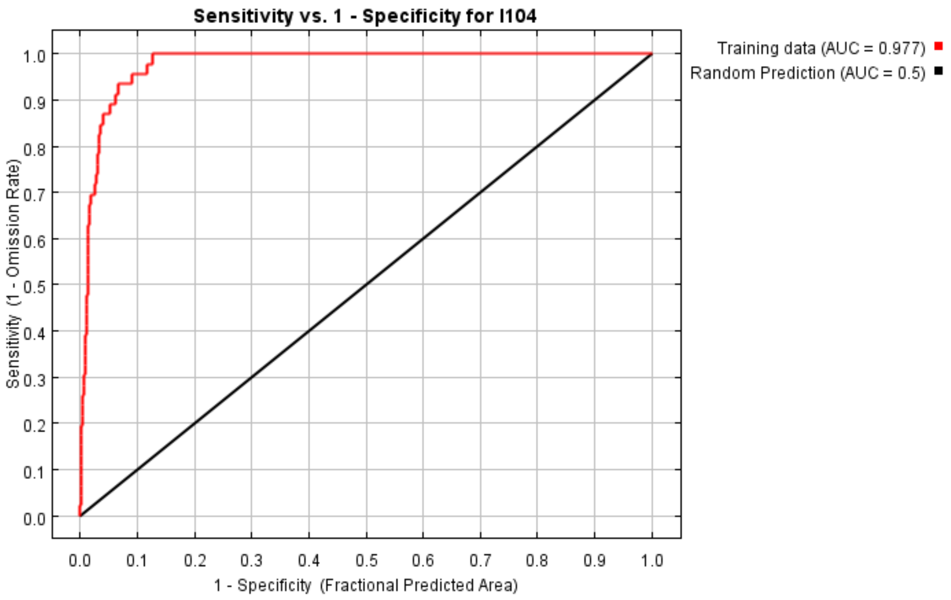


Figure S2d. The ROC curve for *Chenopodium ambrosioides* under current climate.

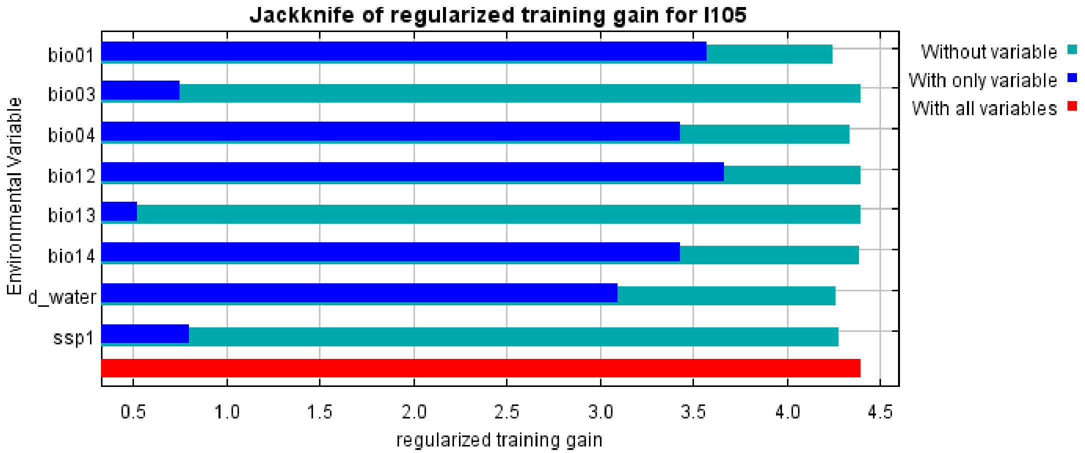


Figure S1e. The Jackknife test results for indicating the relative contribution of environmental varibales for *Coronopus didymus*

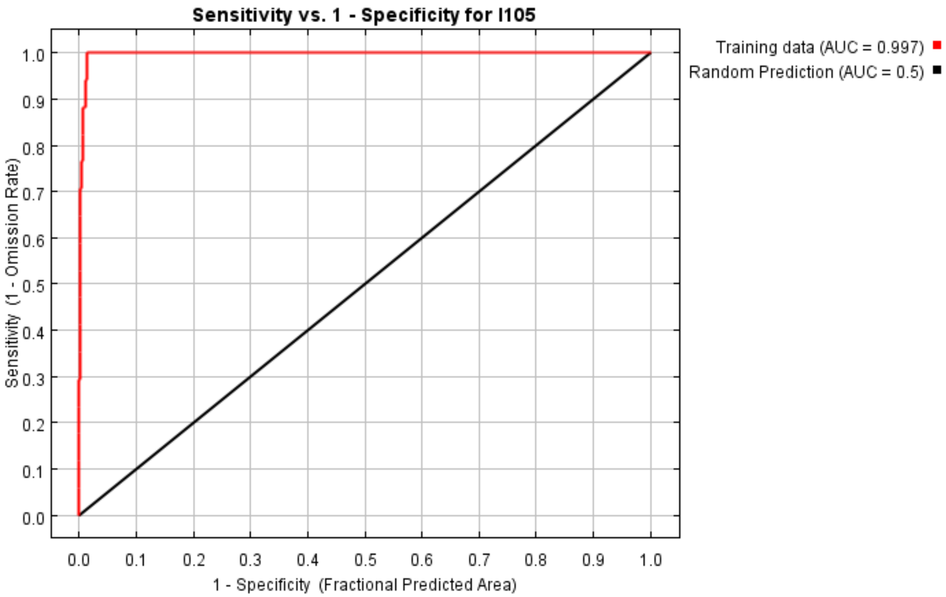


Figure S2e. The ROC curve for *Coronopus didymus* under current climate.

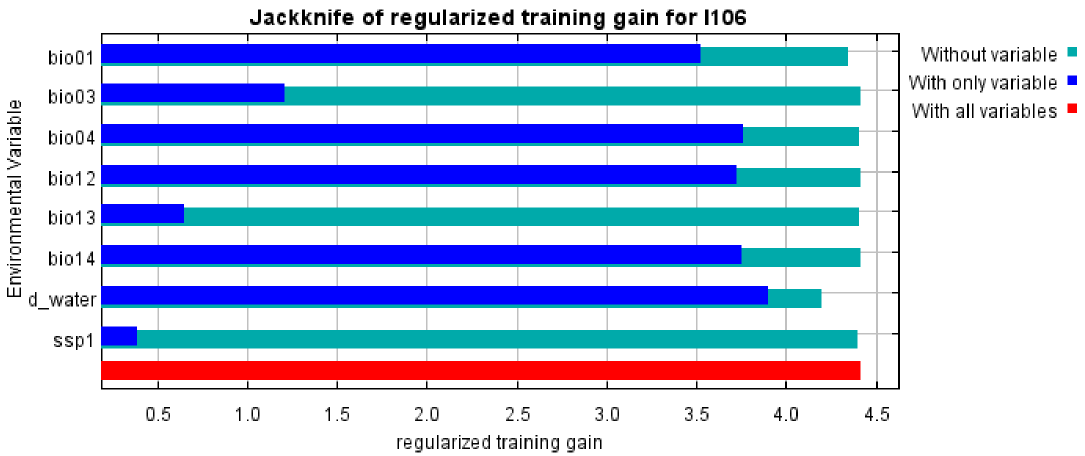


Figure S1f. The Jackknife test results for indicating the relative contribution of environmental varibales for *Gnaphalium calviceps*

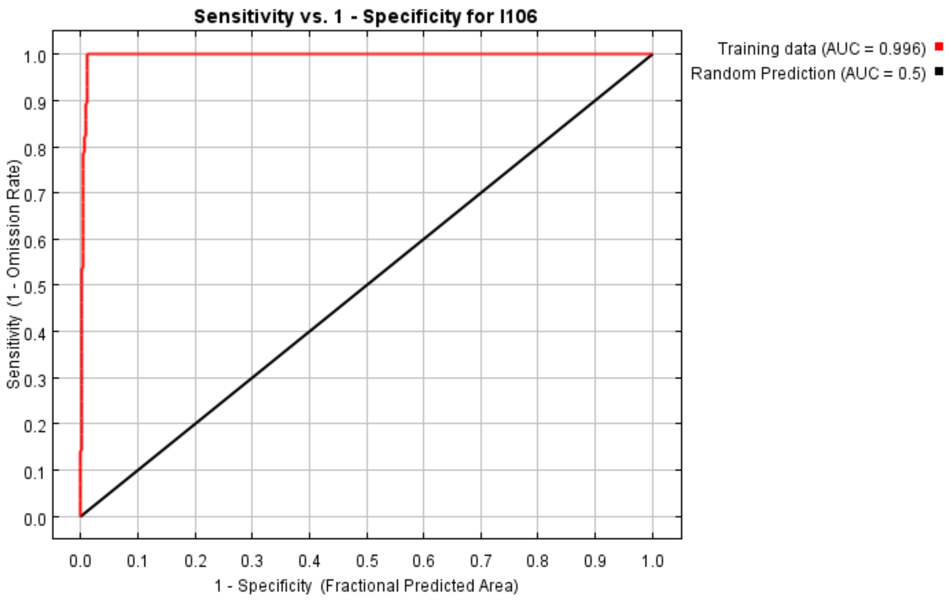


Figure S2f. The ROC curve for *Gnaphalium calviceps*under current climate.

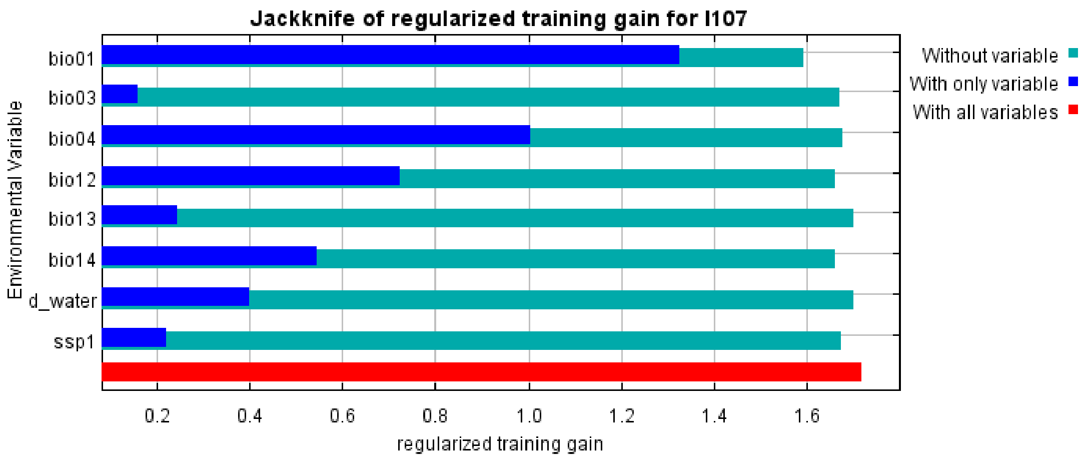


Figure S1g. The Jackknife test results for indicating the relative contribution of environmental varibales for *Lolium multiflorum* var. *multiflorum*

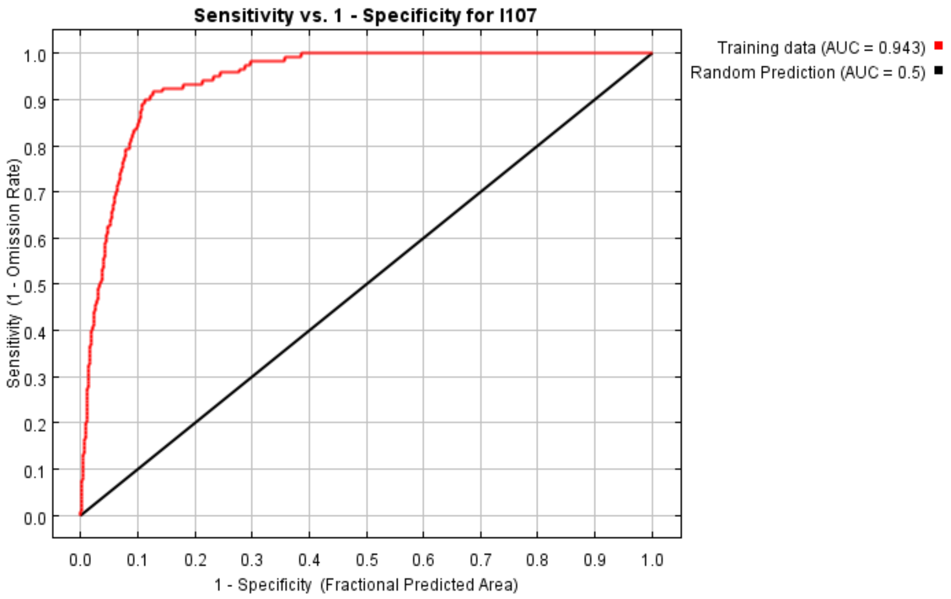


Figure S2g. The ROC curve for *Lolium multiflorum* var *multiflorum* under current climate.

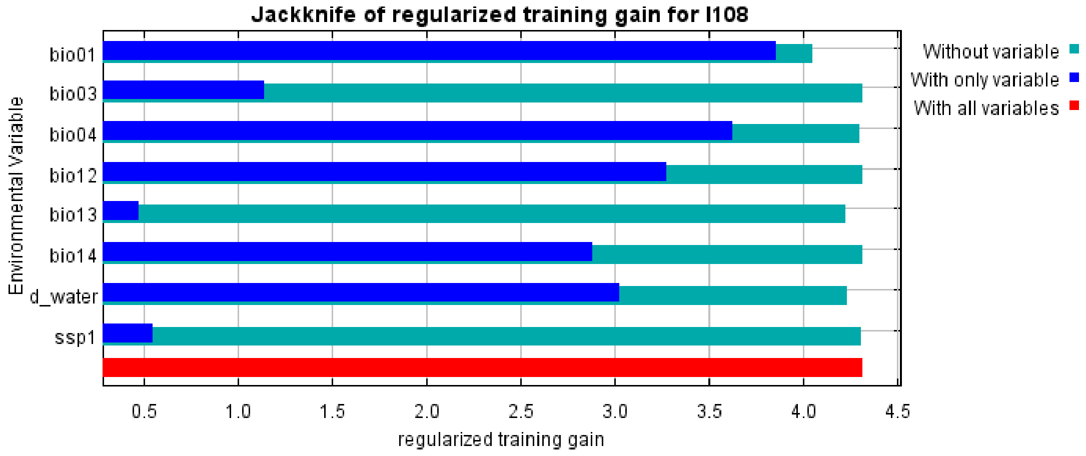


Figure S1h. The Jackknife test results for indicating the relative contribution of environmental varibales for *Modiola caroliniana*

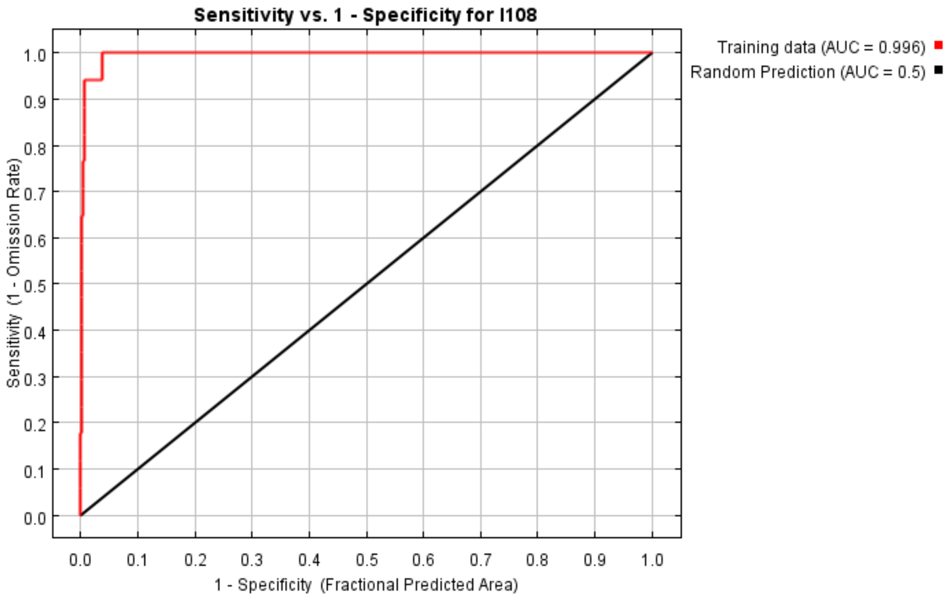


Figure S2h. The ROC curve for *Modiola caroliniana* under current climate.

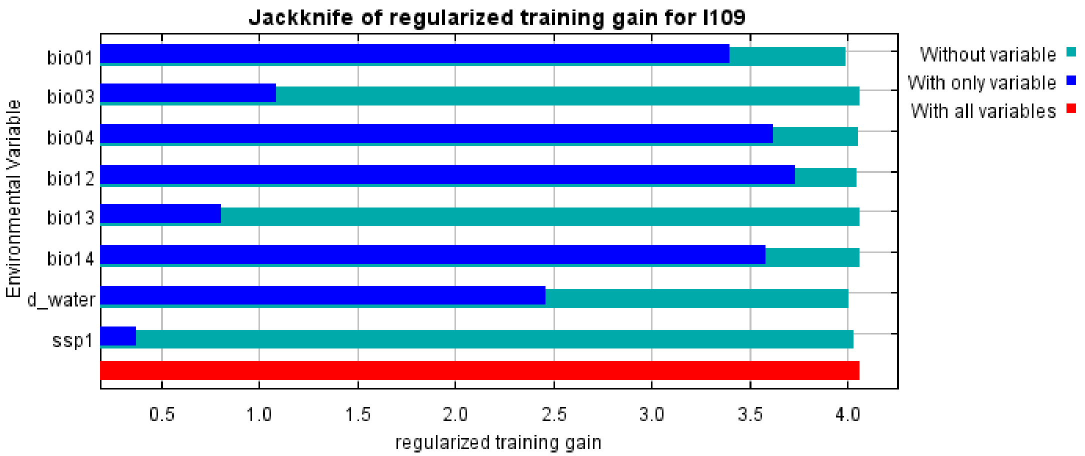


Figure S1i. The Jackknife test results for indicating the relative contribution of environmental varibales for *Oenothera laciniata*

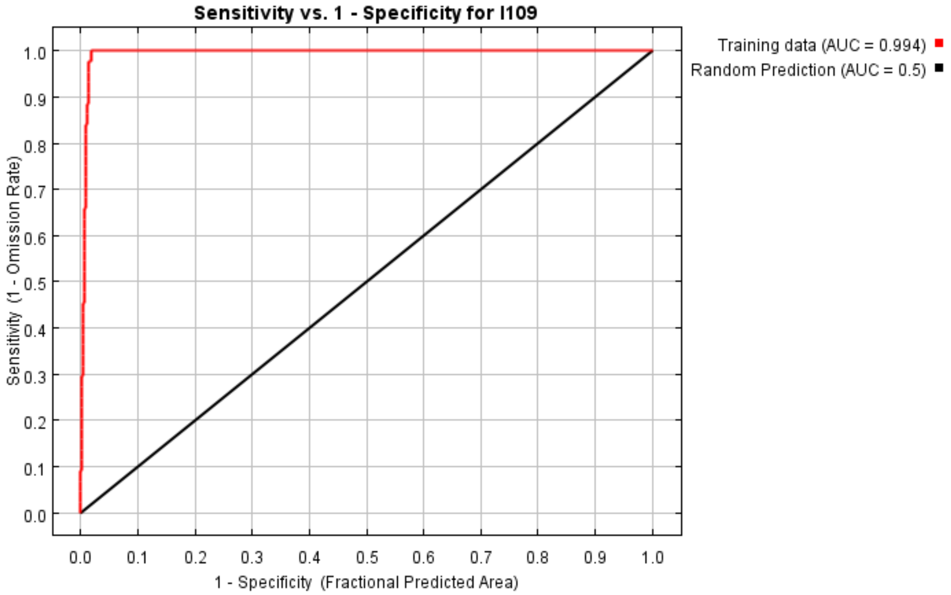


Figure S2i. The ROC curve for *Oenothera laciniate* under current climate.

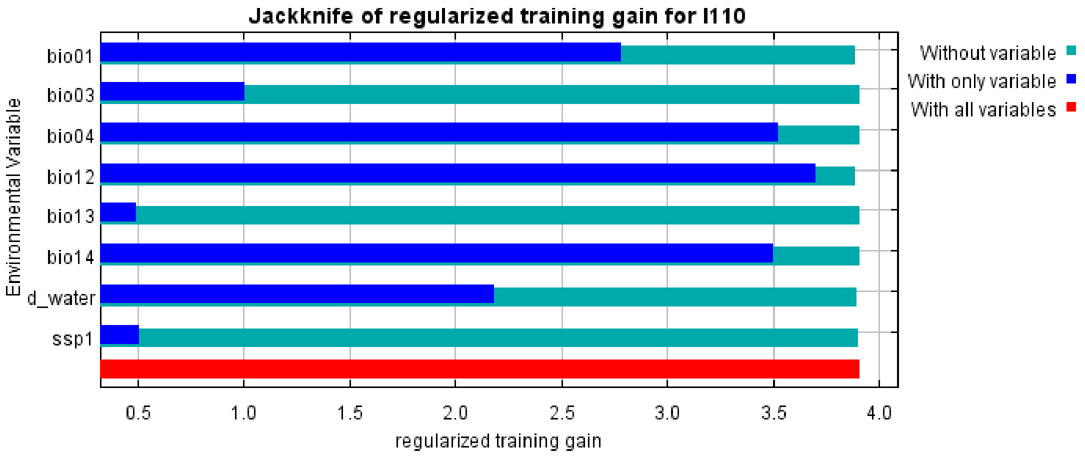


Figure S1j. The Jackknife test results for indicating the relative contribution of environmental varibales for *Paspalum dilatatum*

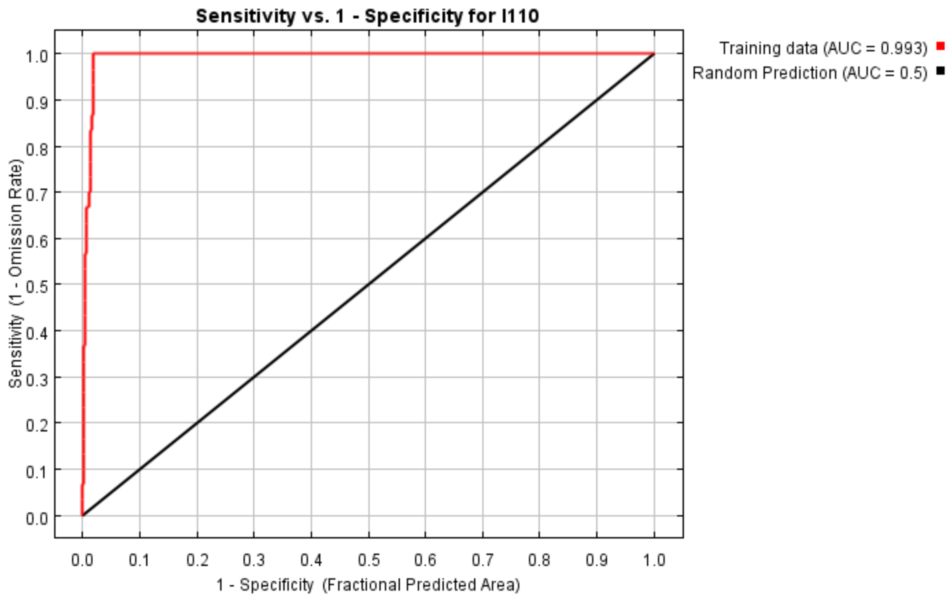


Figure S2j. The ROC curve for *Paspalum dilatatum* under current climate.

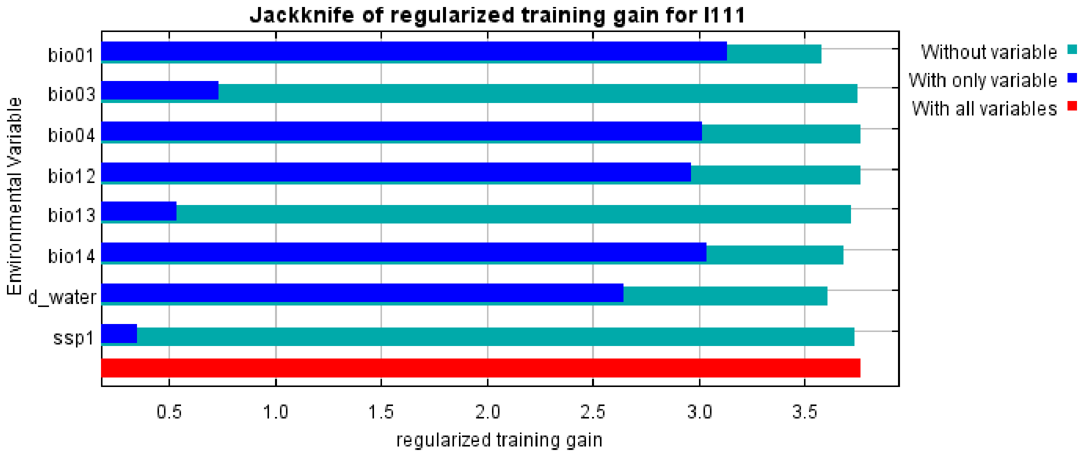


Figure S1k. The Jackknife test results for indicating the relative contribution of environmental varibales for *Sida rhombifolia*

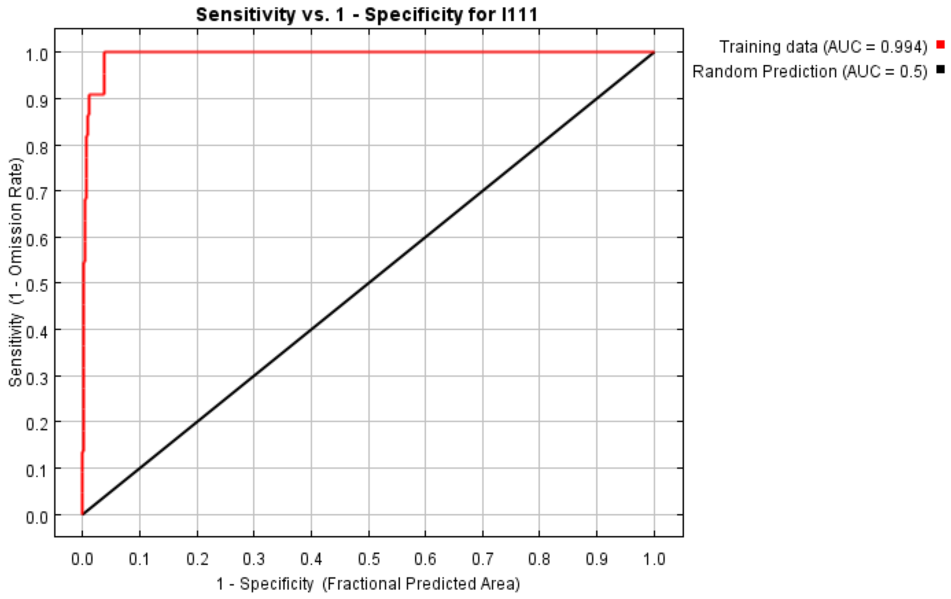


Figure S2k. The ROC curve for *Sida rhombifolia* under current climate.

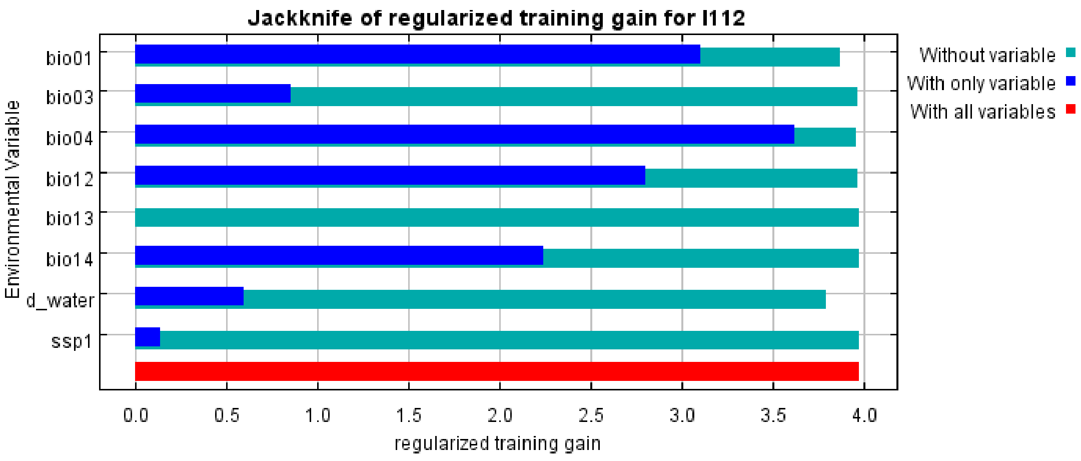


Figure S1l. The Jackknife test results for indicating the relative contribution of environmental varibales for *Silene gallica* var. *gallica*

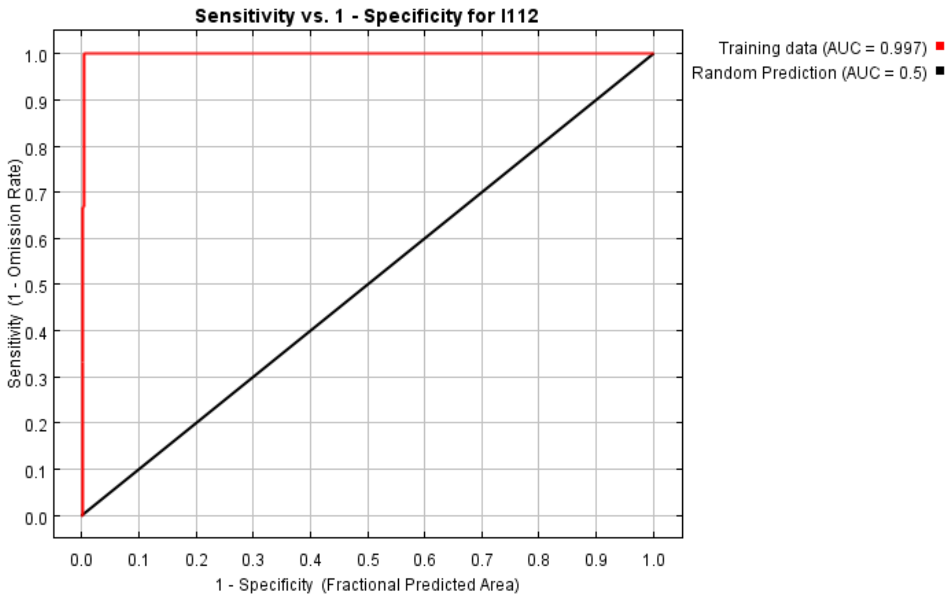


Figure S2l. The ROC curve for *Silene gallica* var. *gallica*under current climate.

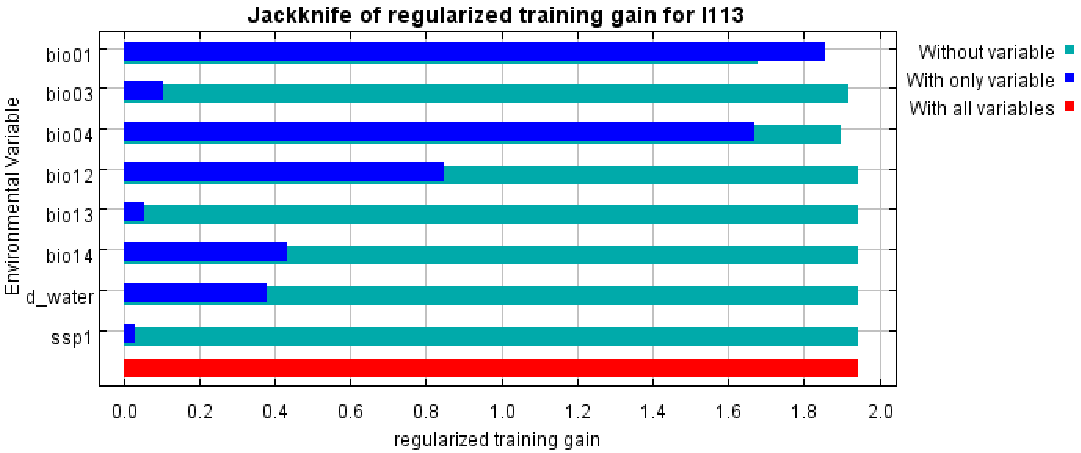


Figure S1m. The Jackknife test results for indicating the relative contribution of environmental varibales for *Sisymbrium officinale*

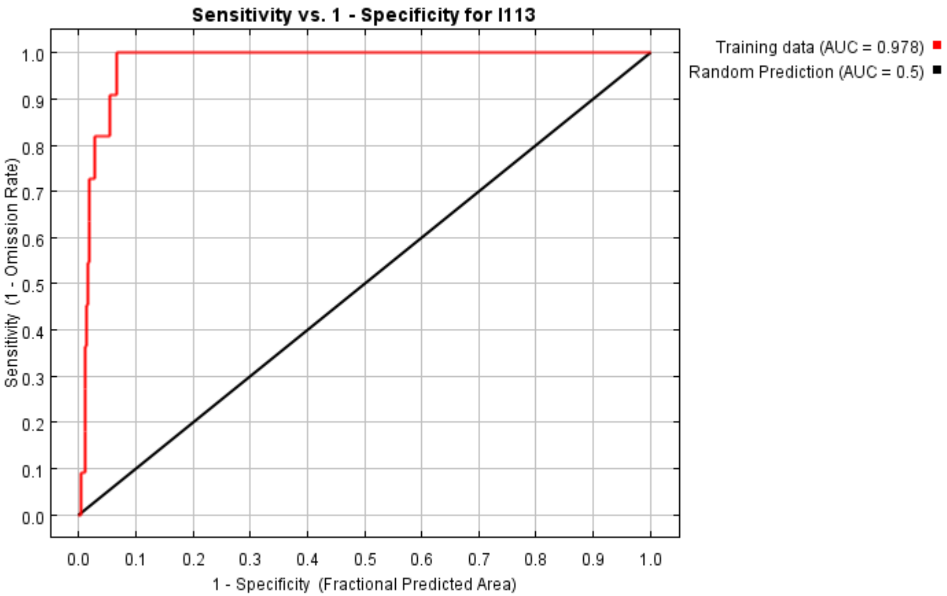


Figure S2m. The ROC curve for *Sisymbrium officinale* under current climate.

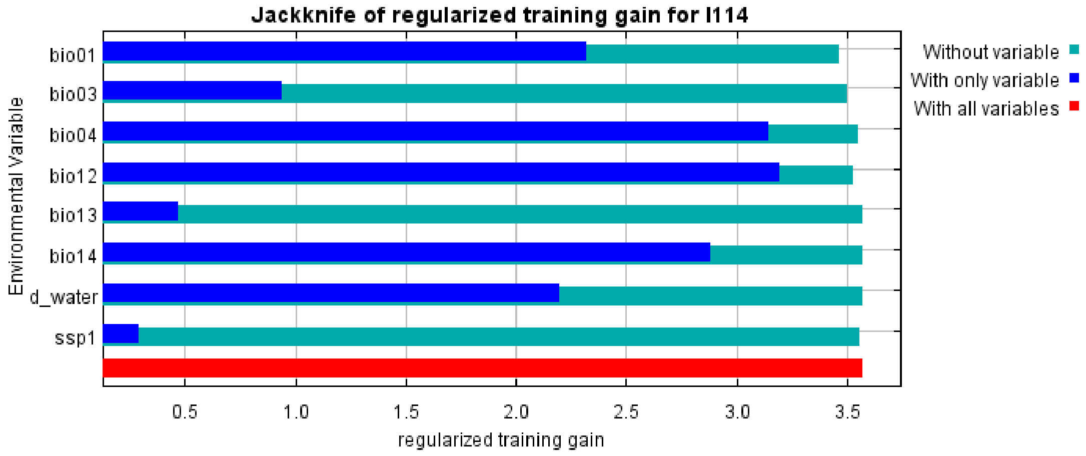


Figure S1n. The Jackknife test results for indicating the relative contribution of environmental varibales for *Sisyrinchium angustifolium*

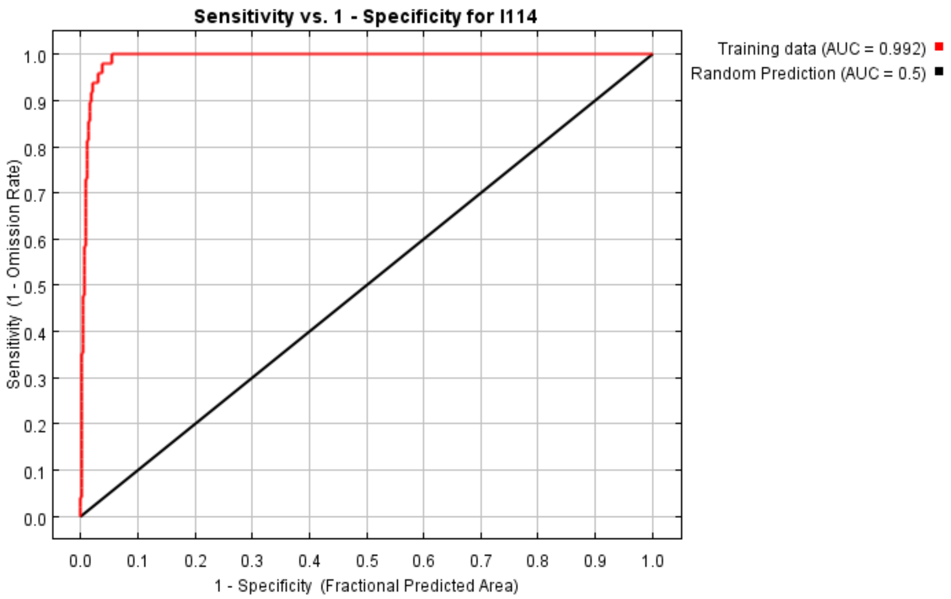


Figure S2n. The ROC curve for *Sisyrinchium angustifolium* under current climate.

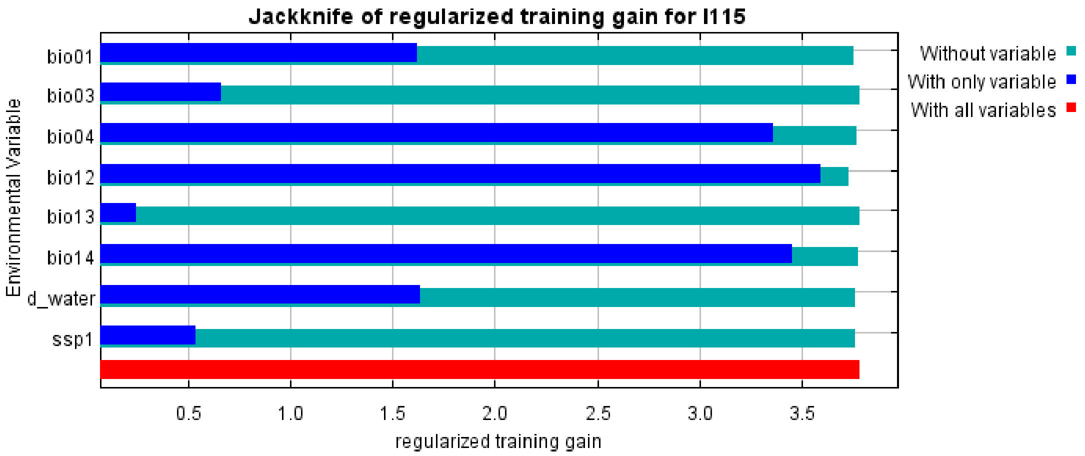


Figure S1o. The Jackknife test results for indicating the relative contribution of environmental varibales for *Spergularia rubra*

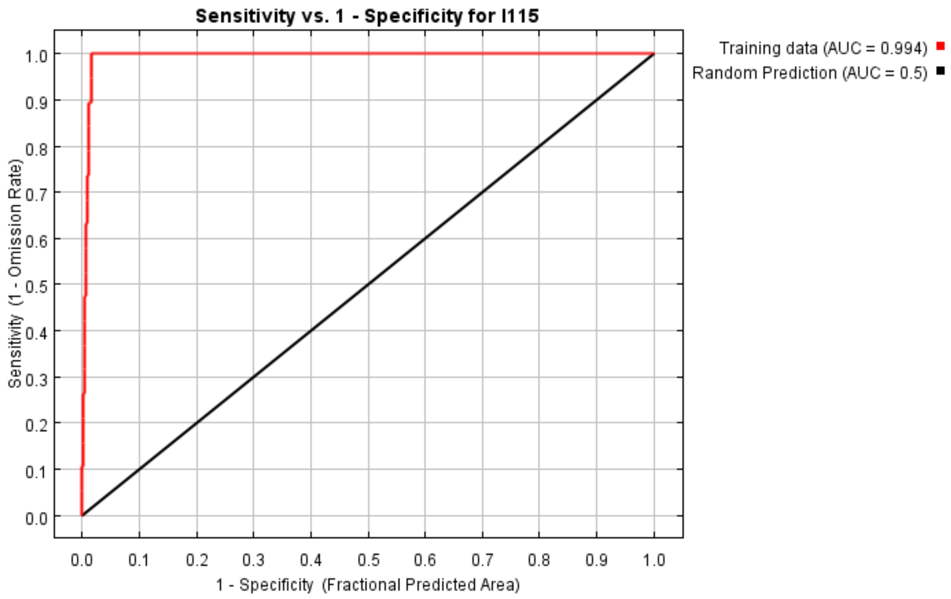


Figure S2o. The ROC curve for *Spergularia rubra* under current climate.

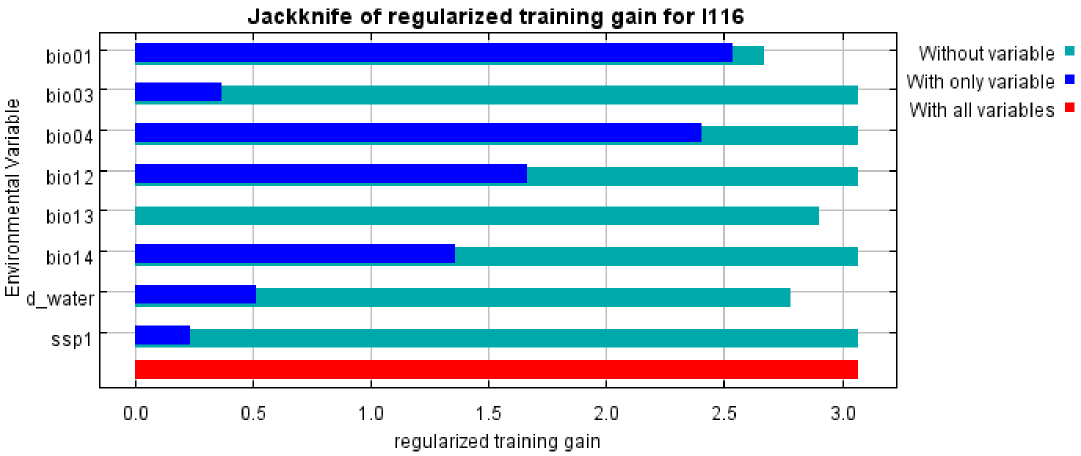


Figure S1p. The Jackknife test results for indicating the relative contribution of environmental varibales for *Malva parviflora*

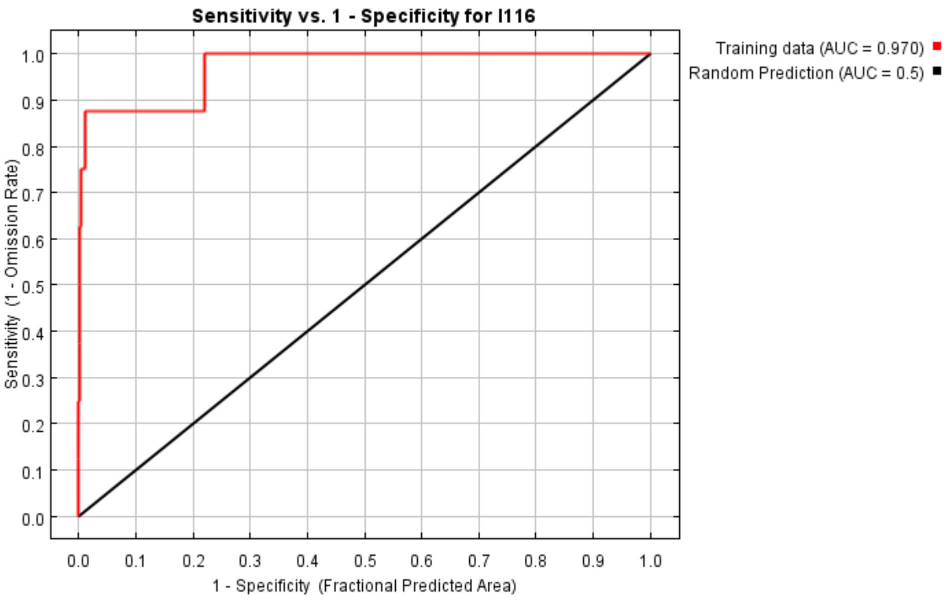


Figure S2p. The ROC curve for *Malva parviflora* under current climate.
