# Supplementary material for: Predicting Impacts of Climate Change on Northward Range Expansion of Invasive Weeds in South Korea
Source: Plants (Basel). 2021 Aug 5;10(8):1604. doi: 10.3390/plants10081604 (PMC8401637; doi:10.3390/plants10081604)
Supplement: Supplementary file 1 [file plants-10-01604-s001.zip › Supplementary figures S3.pdf]

Figure S3a-p. Potential distribution of invasive weeds in South Korea. S3a, *Apium leptophyllum*; S3b, *Astragalus sinicus*; S3c, *Bromus unioloides*; S3d, *Chenopodium ambrosioides*; S3e, *Coronopus didymus*; S3f, *Gnaphalium calviceps*; S3g, *Lolium multiflorum* var. *multiflorum*; S3h, *Modiola carolinian a*; S3i, *Oenothera laciniate*; S3j, *Paspalum dilatatum*; S3k, *Sida rhombifolia*; S3l, *Silene gallica* var. *gallica*; S3m, *Sisymbrium officinale*; S3n, *Sisyrinchium angustifolium*; S3o, *Spergularia* rubra; S3p, *Malva parviflora*.

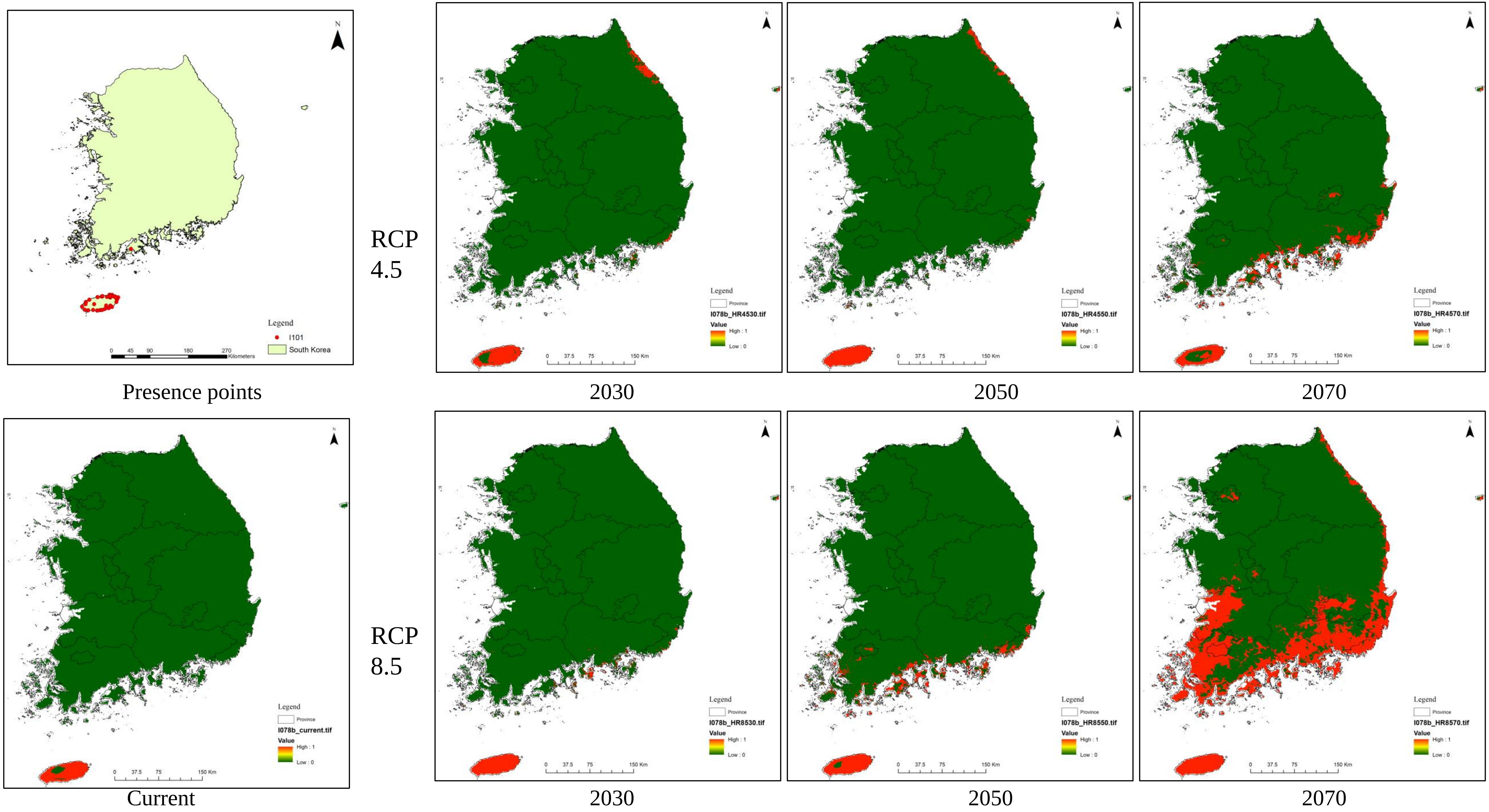

Figure S3a. Potential distribution of *Apium leptophyllum* (솔잎미나리) (I101) under RCP 4.5 and RCP 8.5.

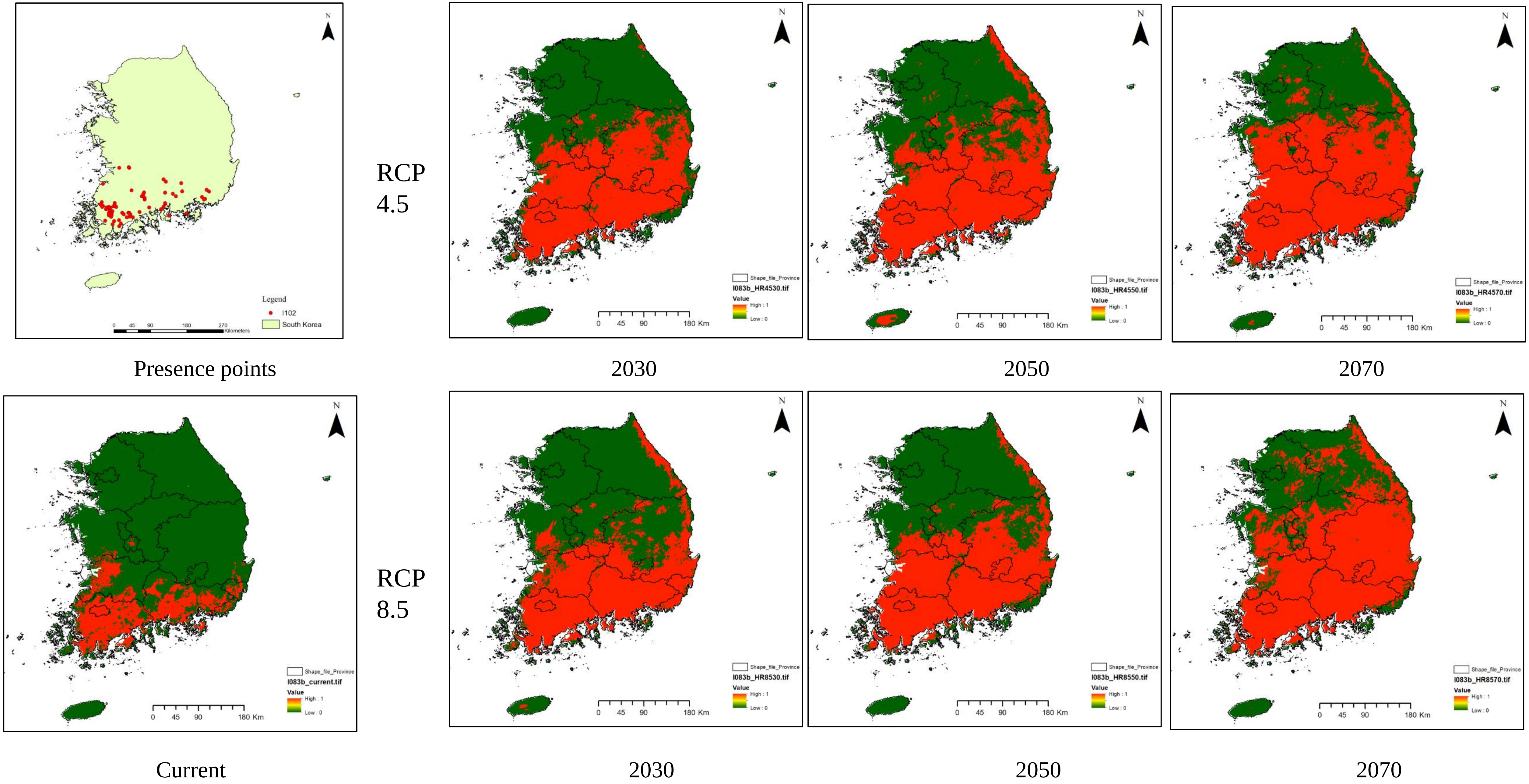

Figure S3b. Potential distribution of *Astragalus sinicus* (자운영) (I102) under RCP 4.5 and RCP 8.5.

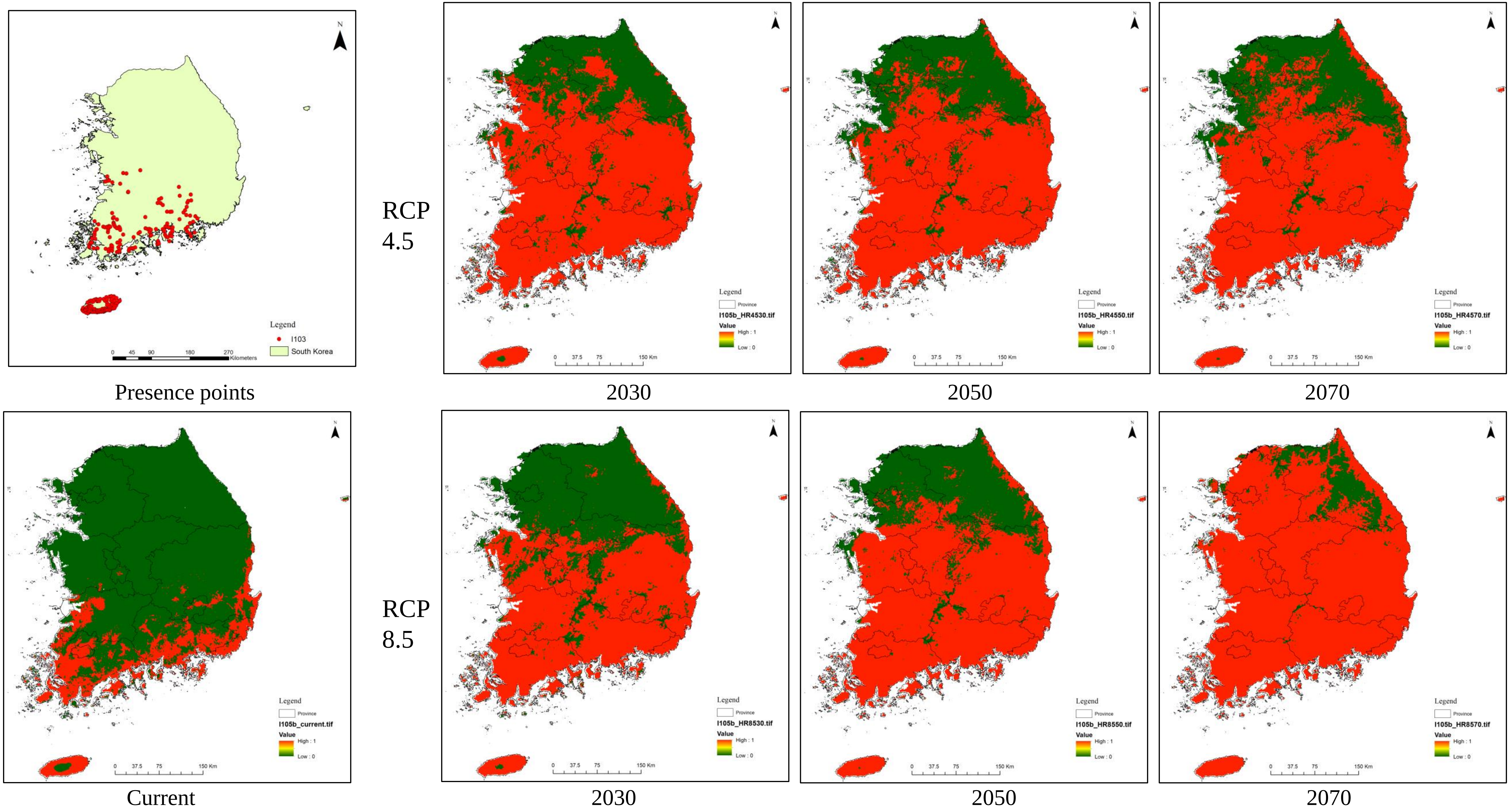

Figure S3c. Potential distribution of *Bromus unioloides* (큰이삭풀) (I103) under RCP 4.5 and RCP 8.5.

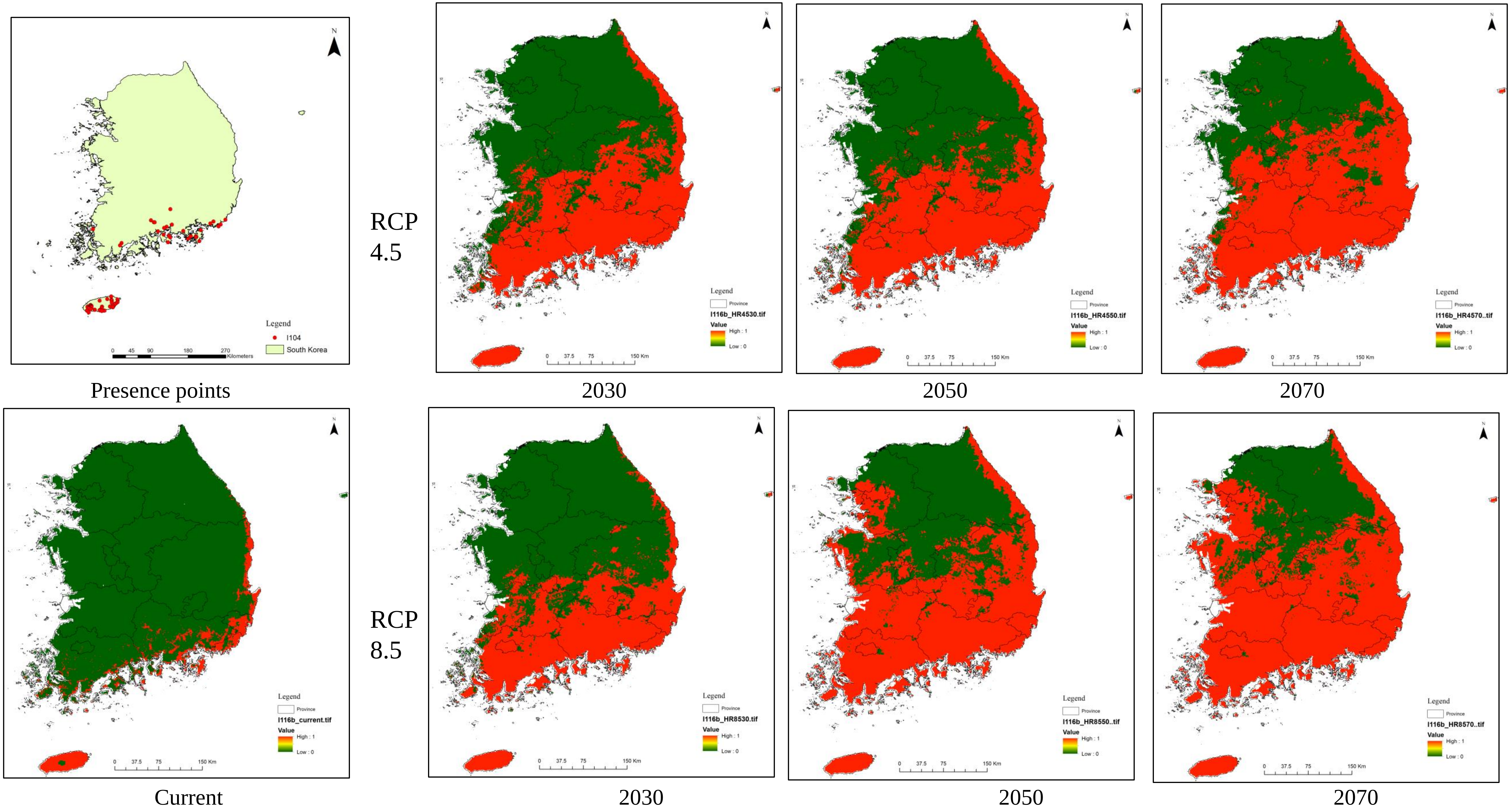

Figure S3d. Potential distribution of *Chenopodium ambrosioides* (양명아주) (I104) under RCP 4.5 and RCP 8.5.

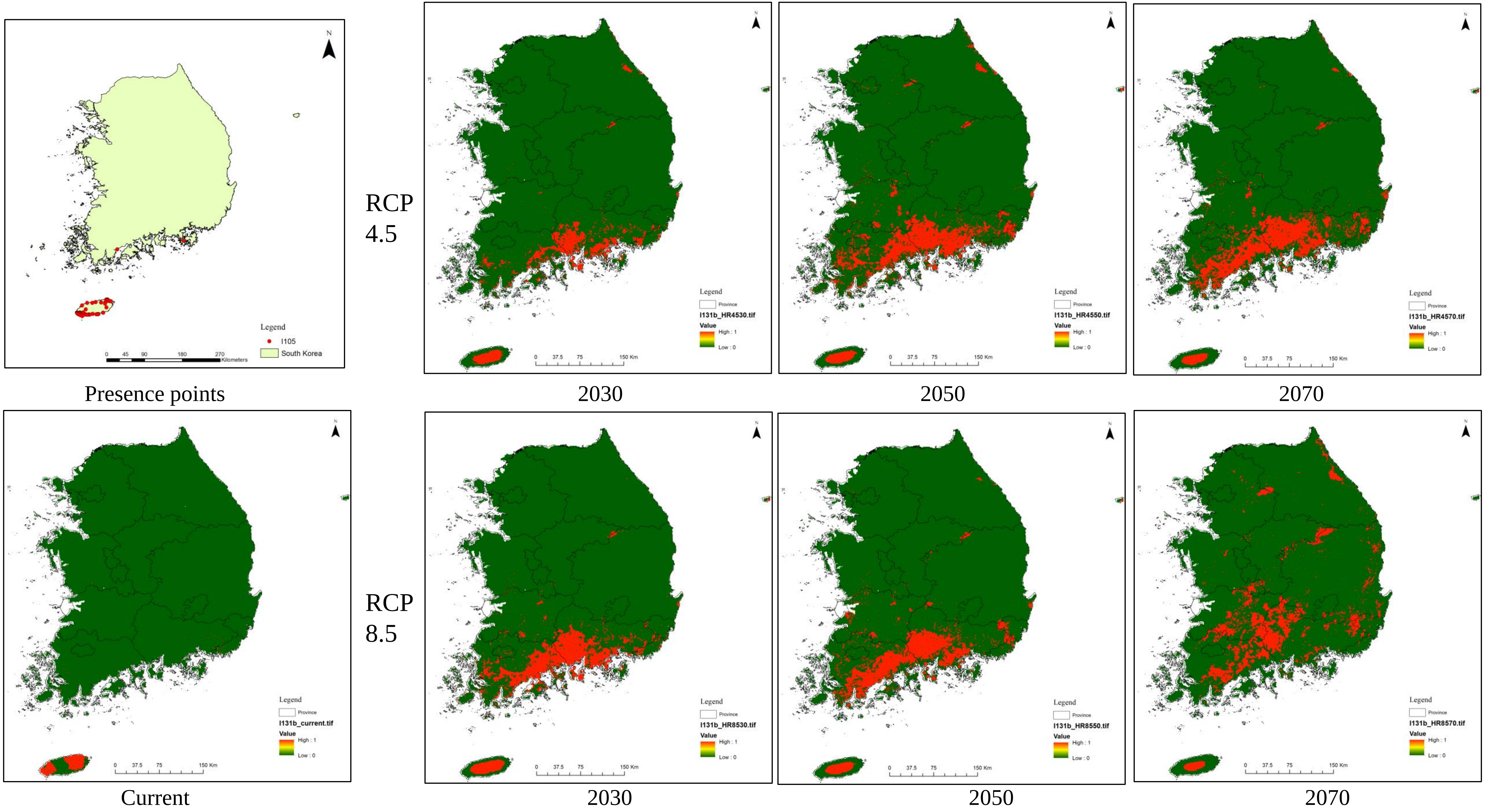

Figure S3e. Potential distribution of *Coronopus didymus* (냄새냉이) (I105) under RCP 4.5 and RCP 8.5.

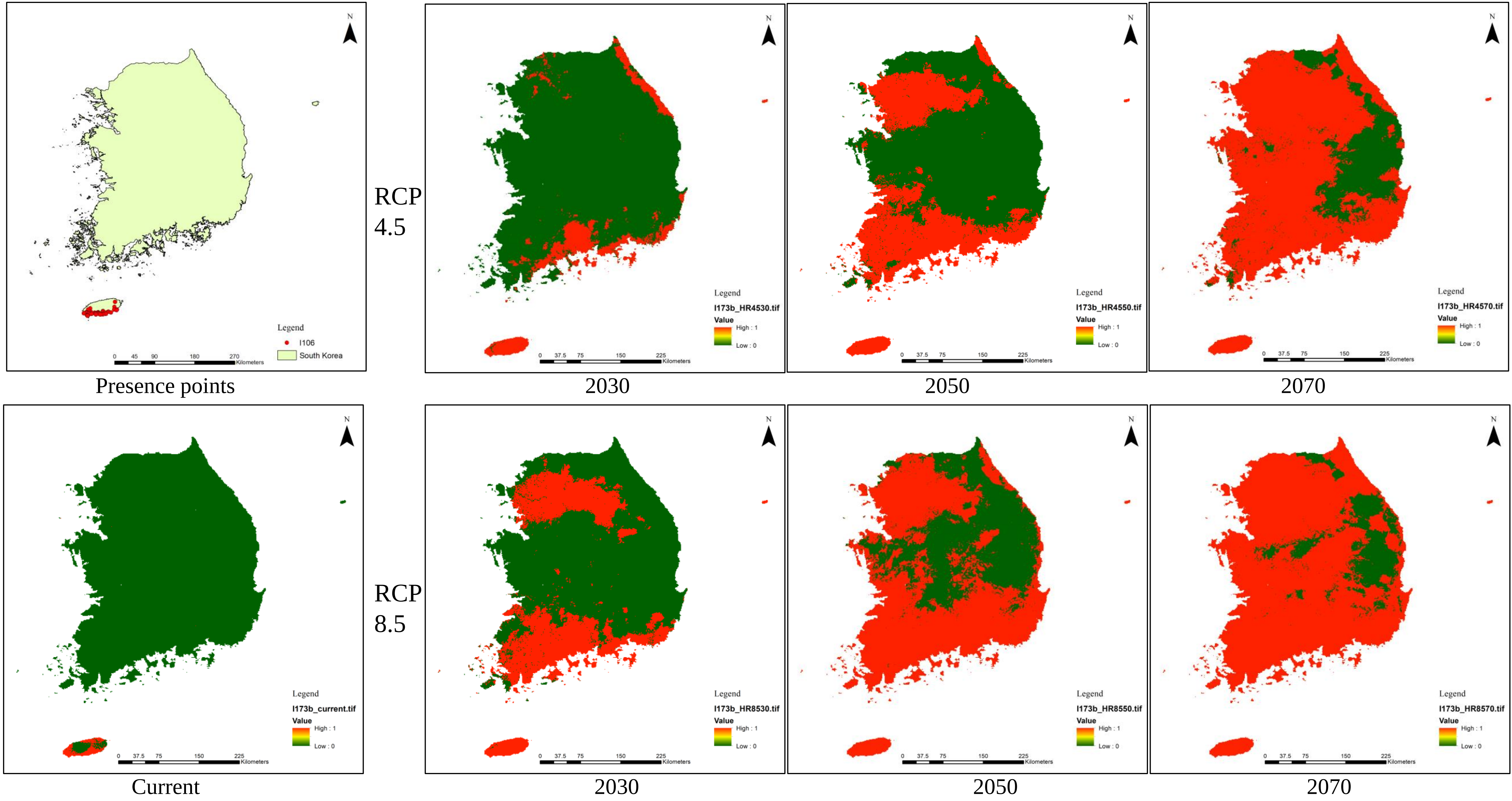

Figure S3f. Potential distribution of *Gnaphalium calviceps* (선풀솜나물) (I106) under RCP 4.5 and RCP 8.5.

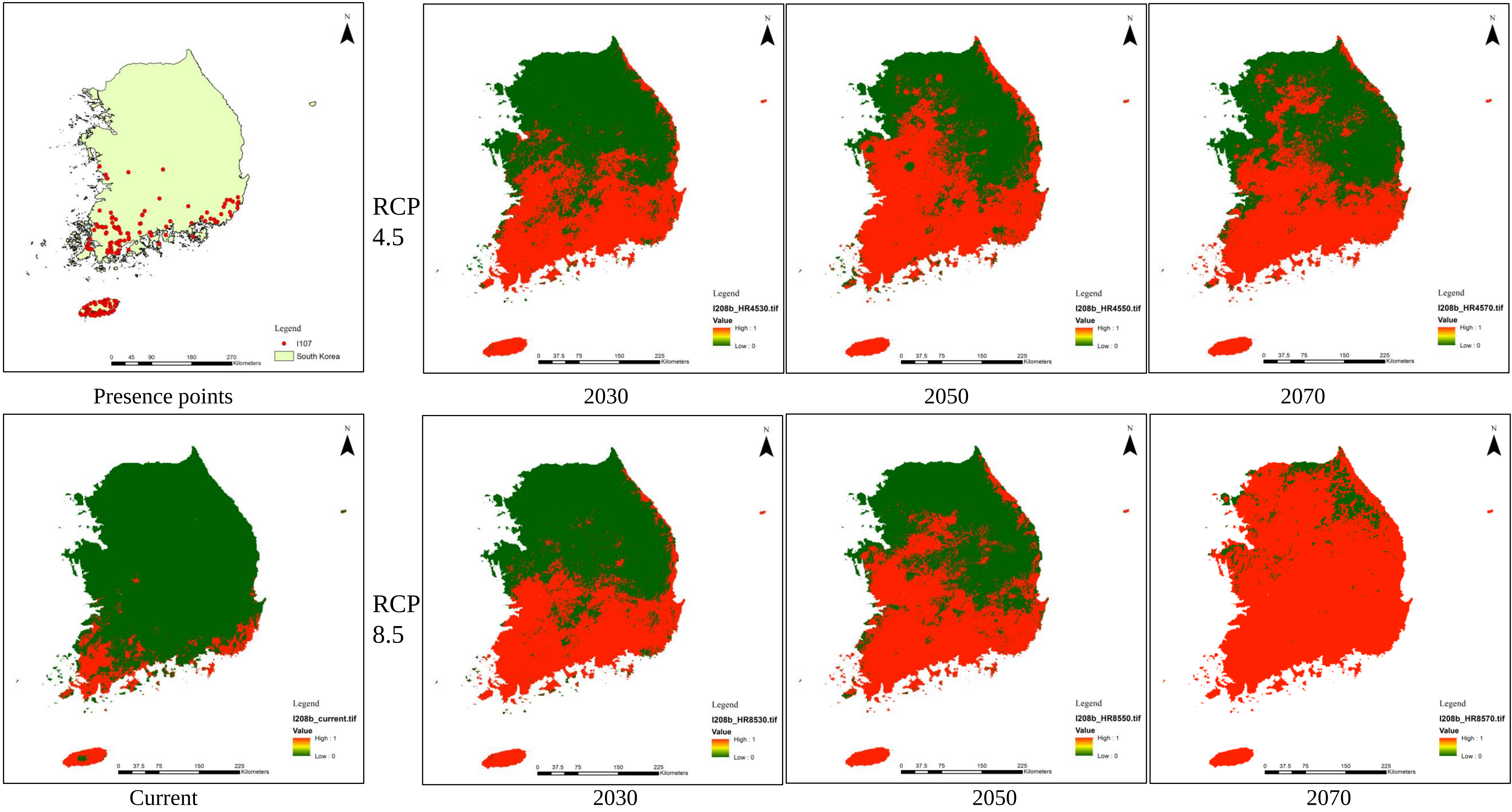

Figure S3g. Potential distribution of *Lolium multiflorum* var. *multiflorum* (I107) under RCP 4.5 and RCP 8.5.

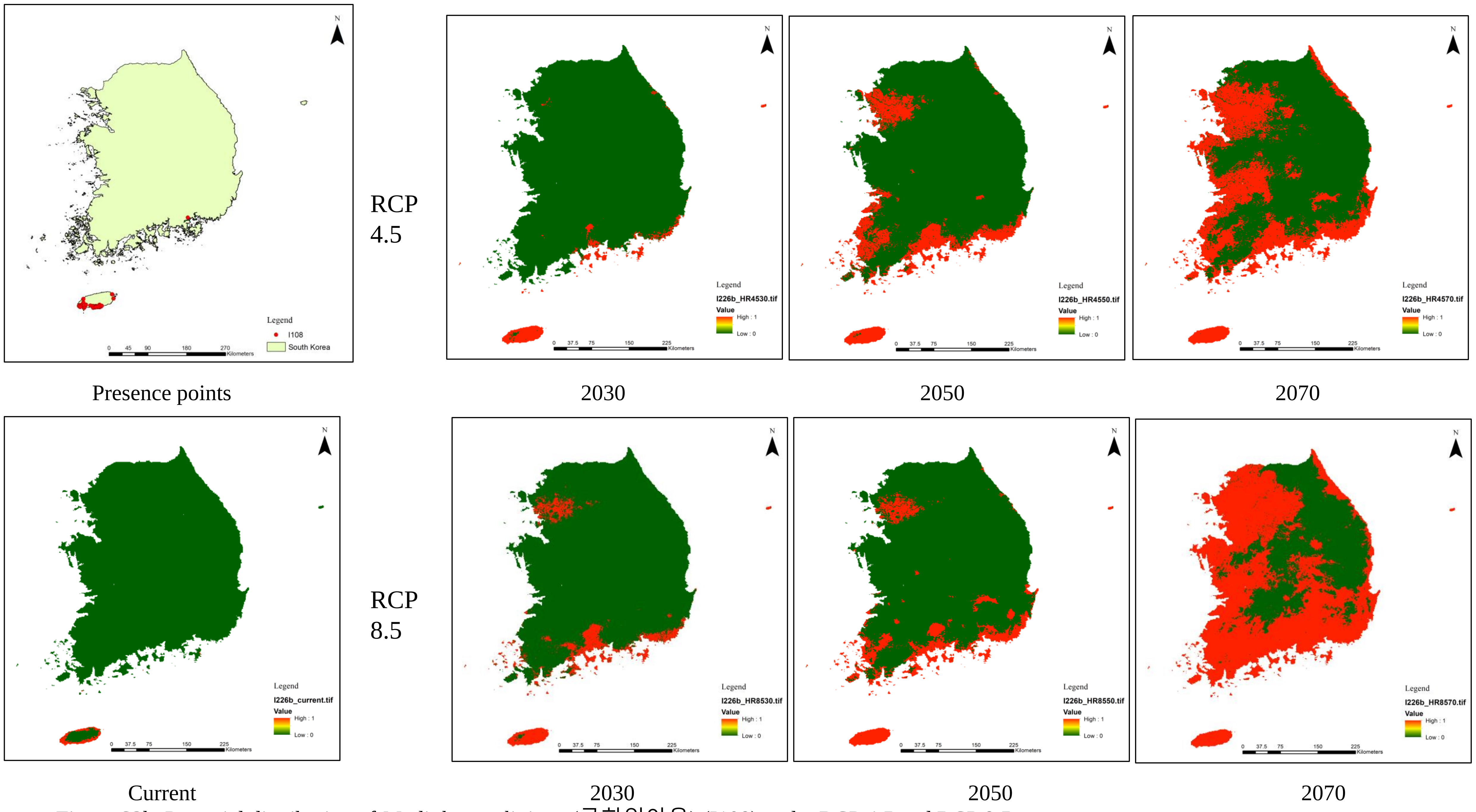

Figure S3h. Potential distribution of *Modiola caroliniana* (국화잎아욱) (I108) under RCP 4.5 and RCP 8.5.

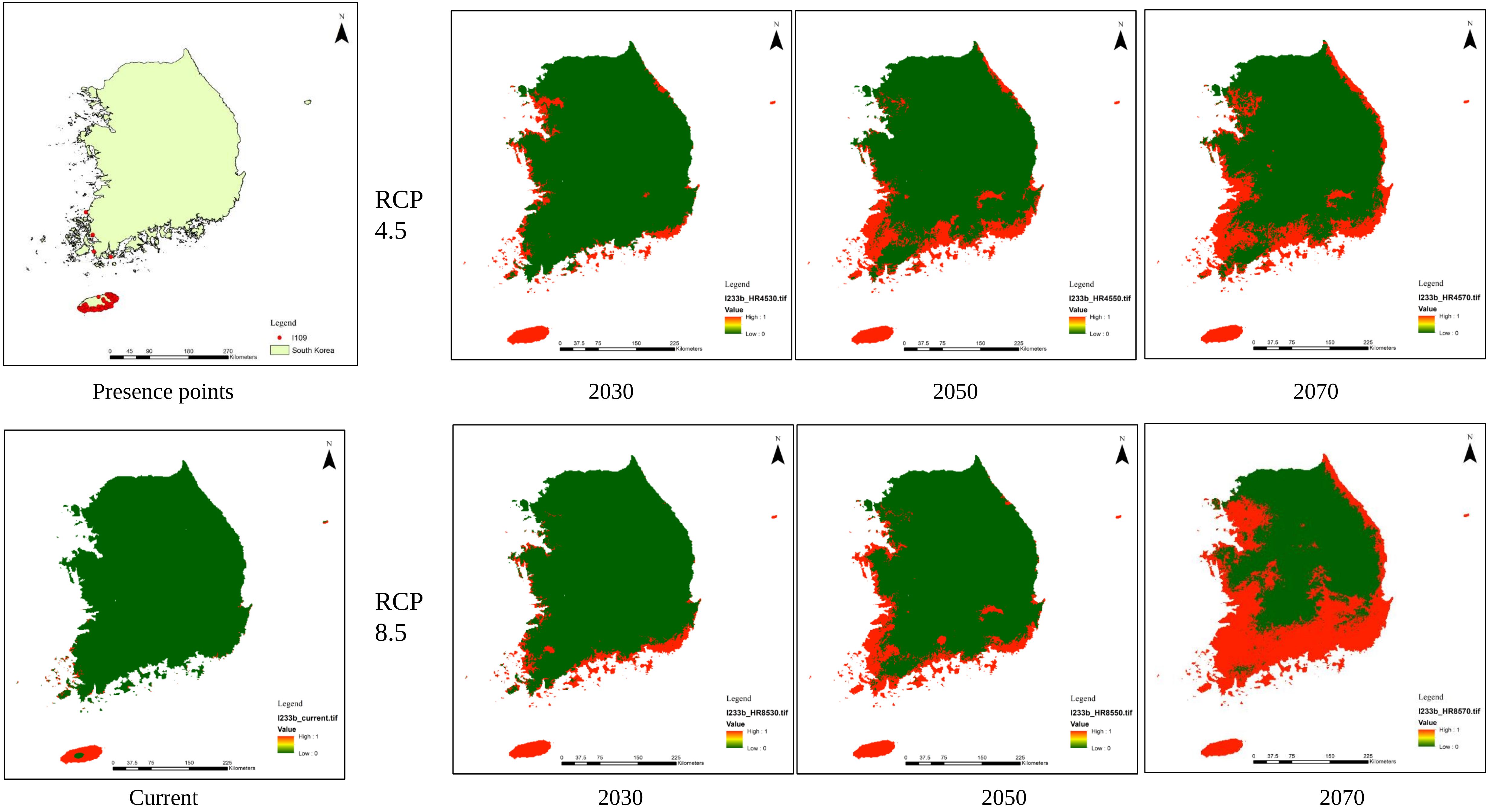

Figure S3i. Potential distribution of *Oenothera laciniata* (애기달맞이꽃) (I109) under RCP 4.5 and RCP 8.5.

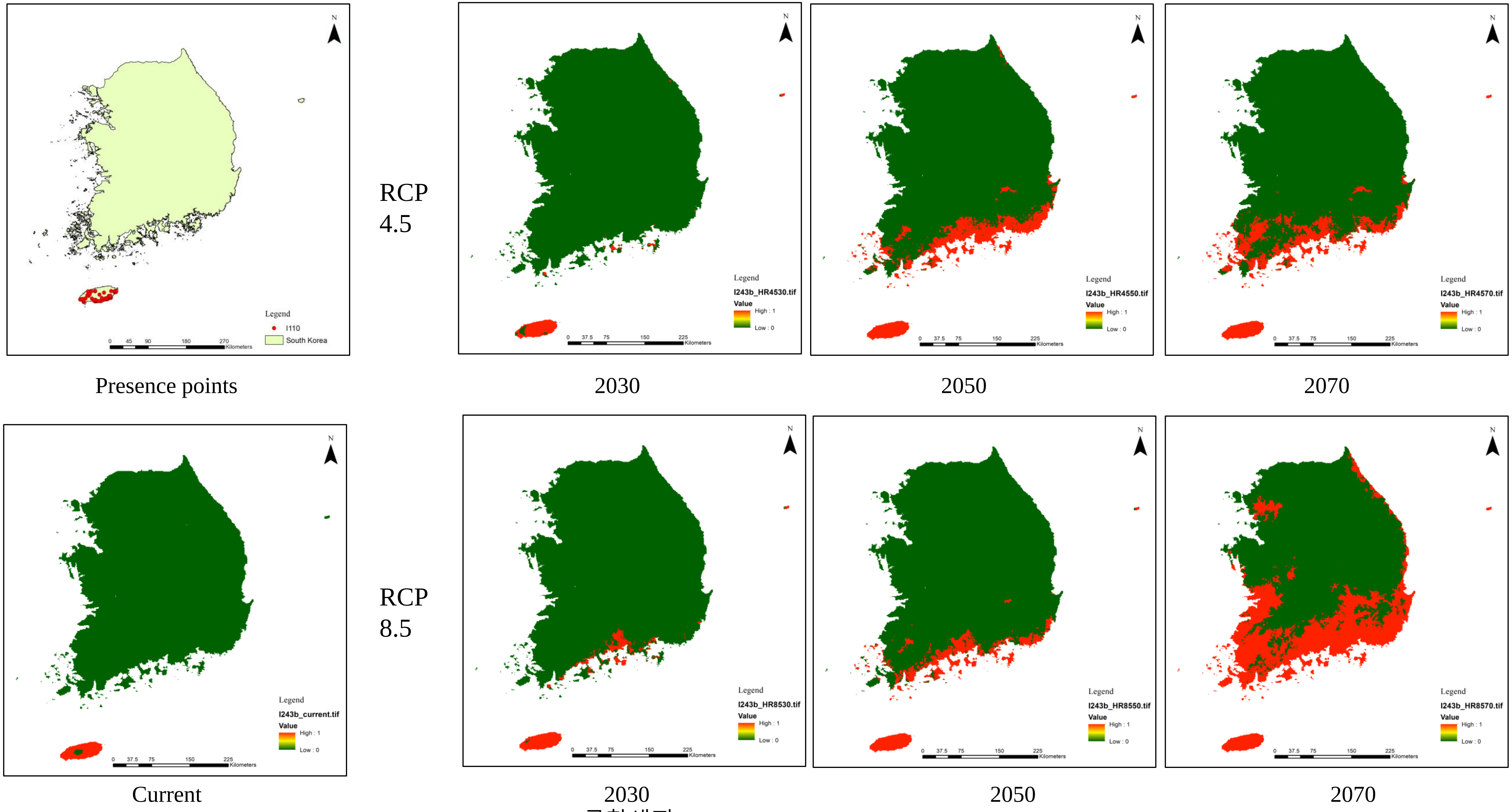

Figure S3j. Potential distribution of *Paspalum dilatatum* (큰참새피) (I110) under RCP 4.5 and RCP 8.5.

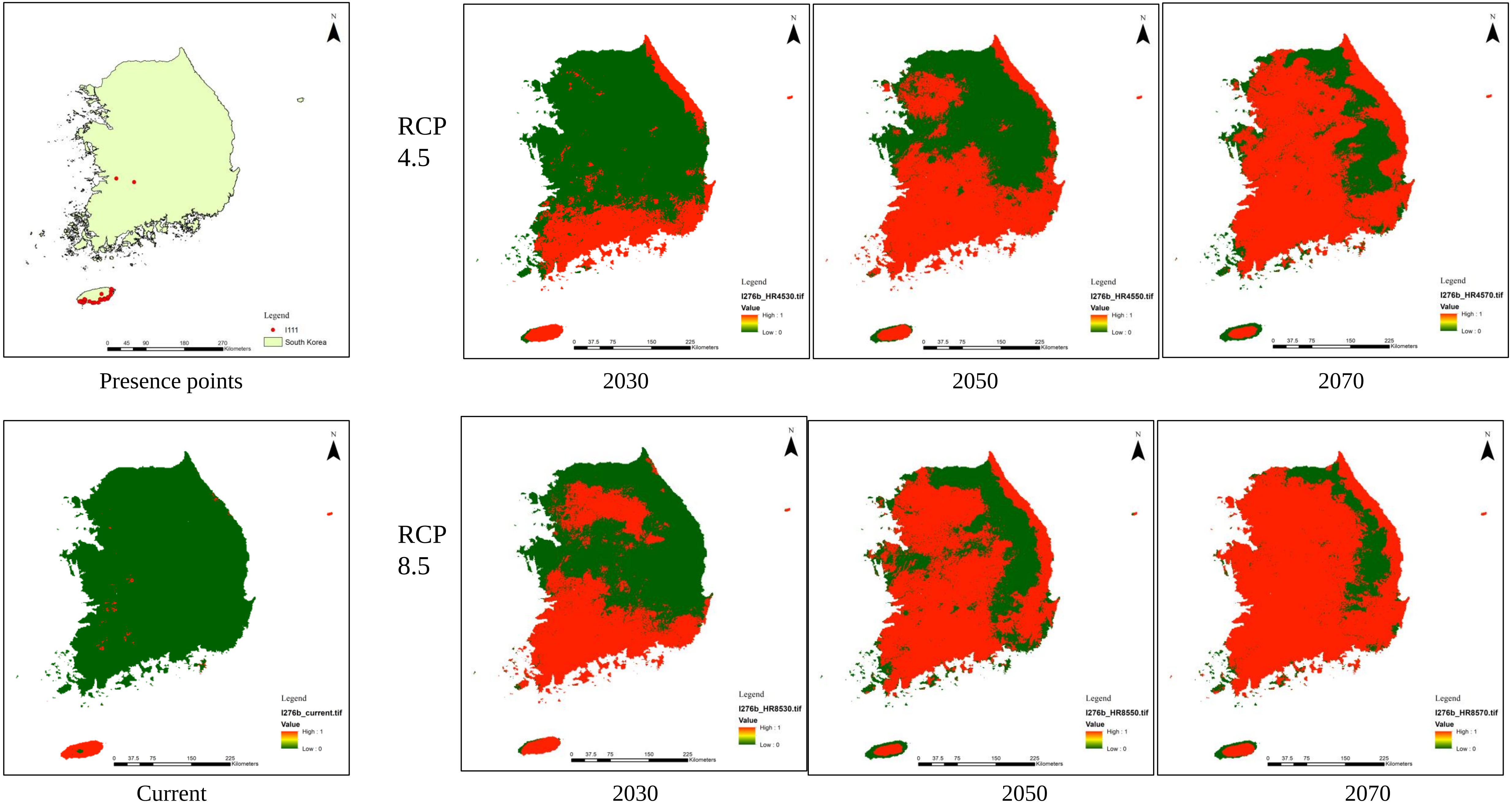

Figure S3k. Potential distribution of *Sida rhombifolia* (나도공단풀) (I111) under RCP 4.5 and RCP 8.5.

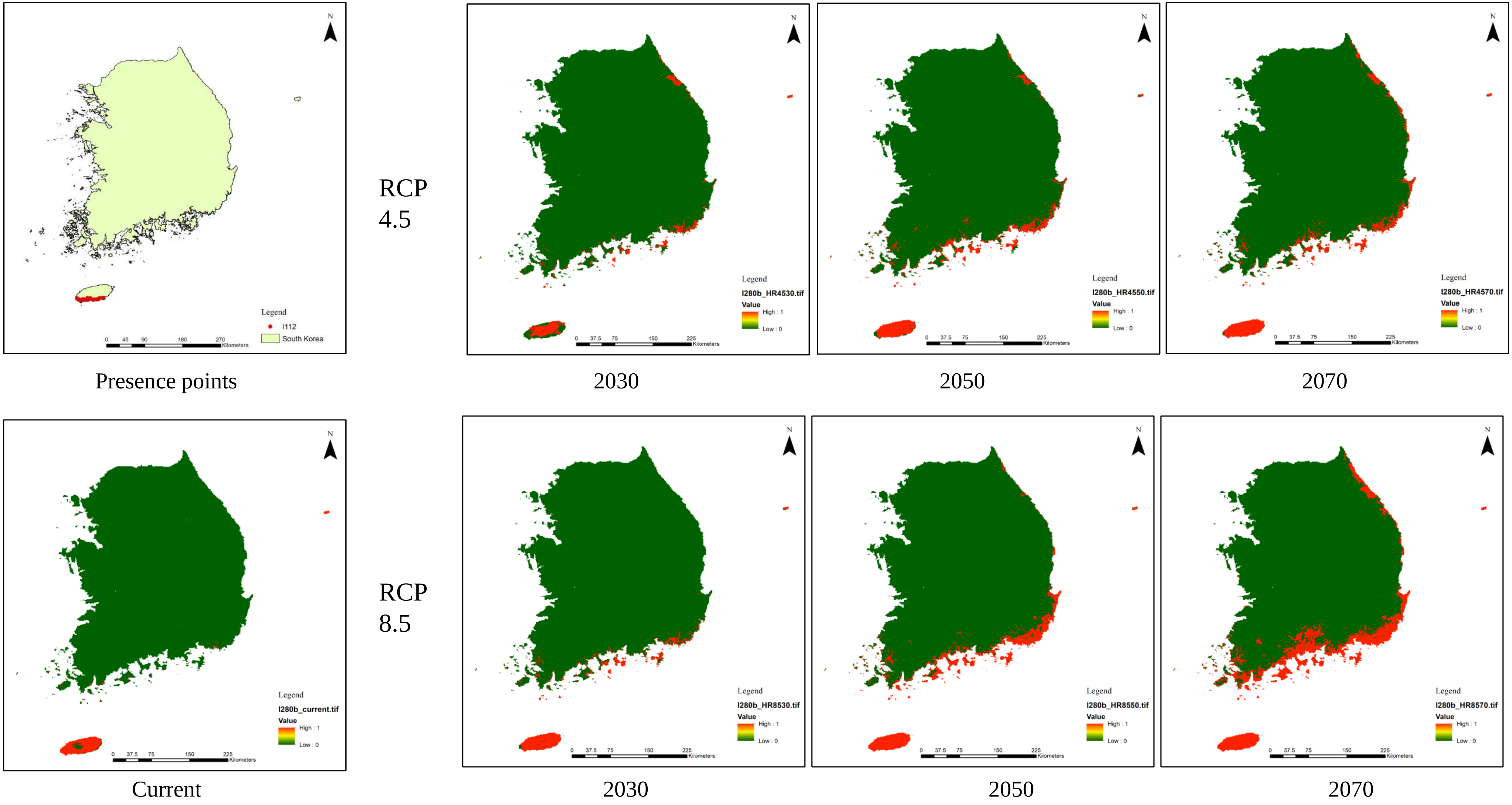

Figure S3l. Potential distribution of *Silene gallica* var. *gallica* (I112) under RCP 4.5 and RCP 8.5.

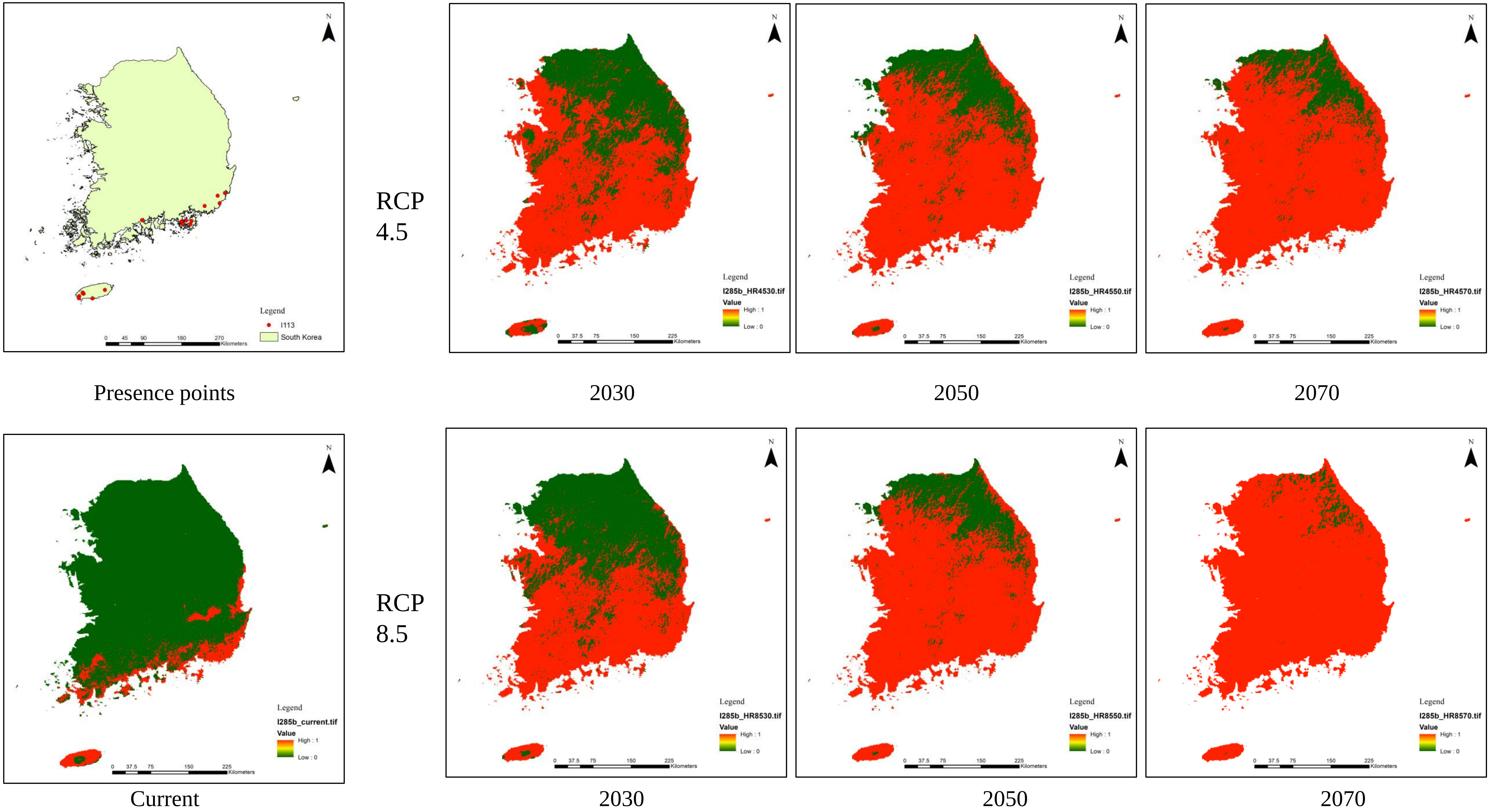

Figure S3m. Potential distribution of *Sisymbrium officinale* var. *leiocarpum* (민유럽장대) (I113) under RCP 4.5 and RCP 8.5.

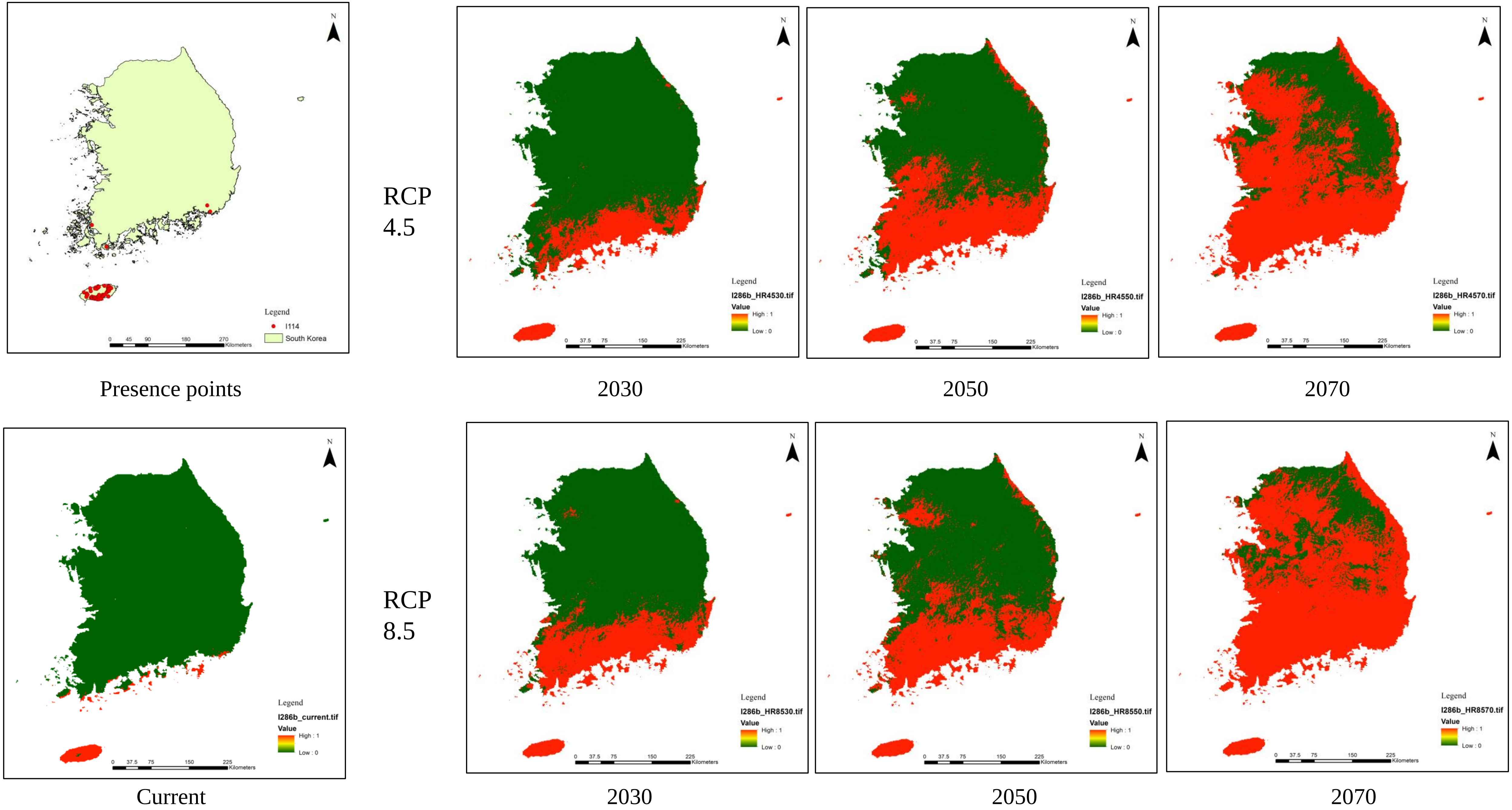

Figure S3n. Potential distribution of *Sisyrinchium angustifolium* (흰등심붓꽃) (I114) under RCP 4.5 and RCP 8.5.

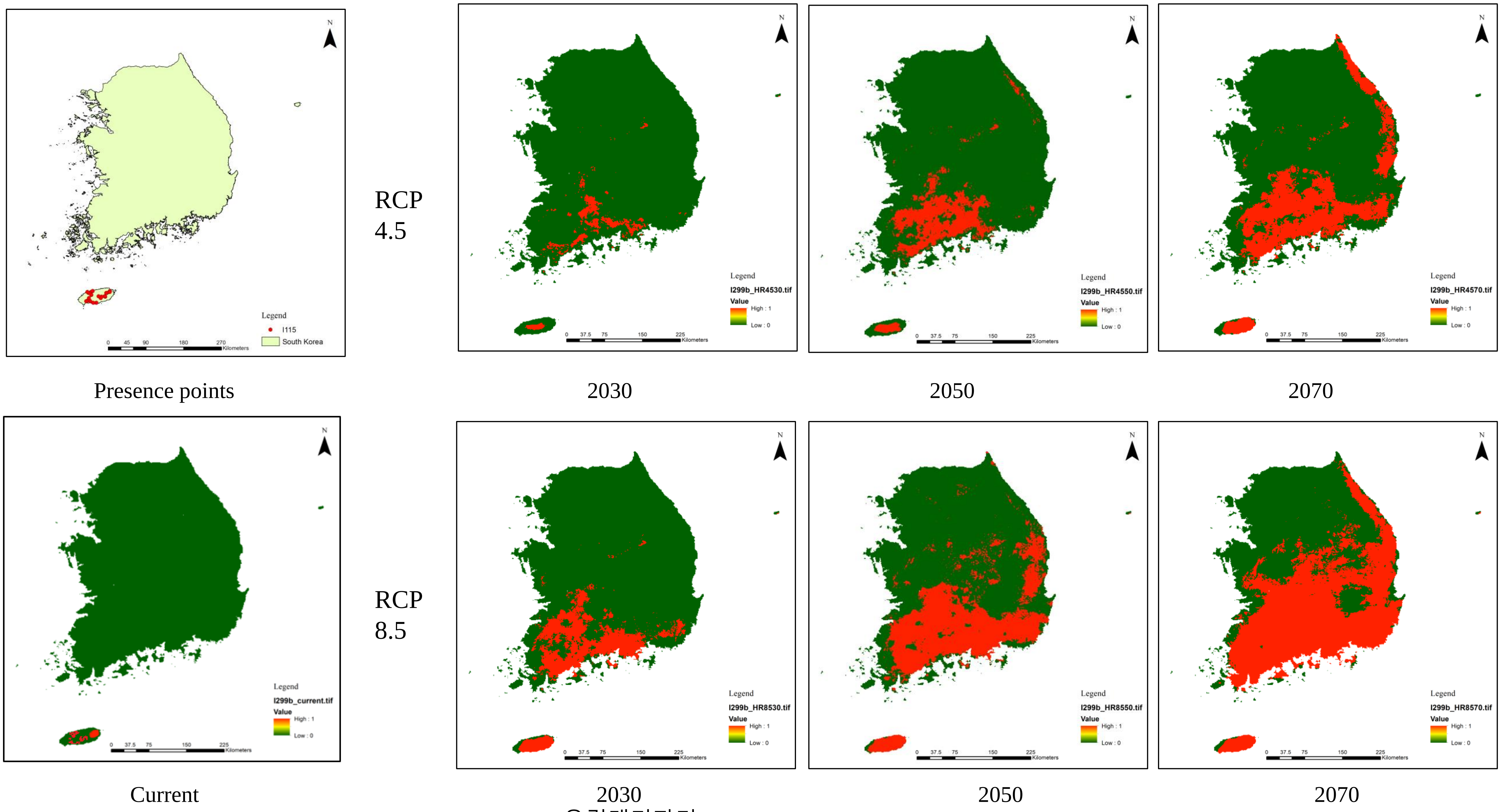

Figure S3o. Potential distribution of *Spergularia rubra* (유럽개미자리) (I115) under RCP 4.5 and RCP 8.5.

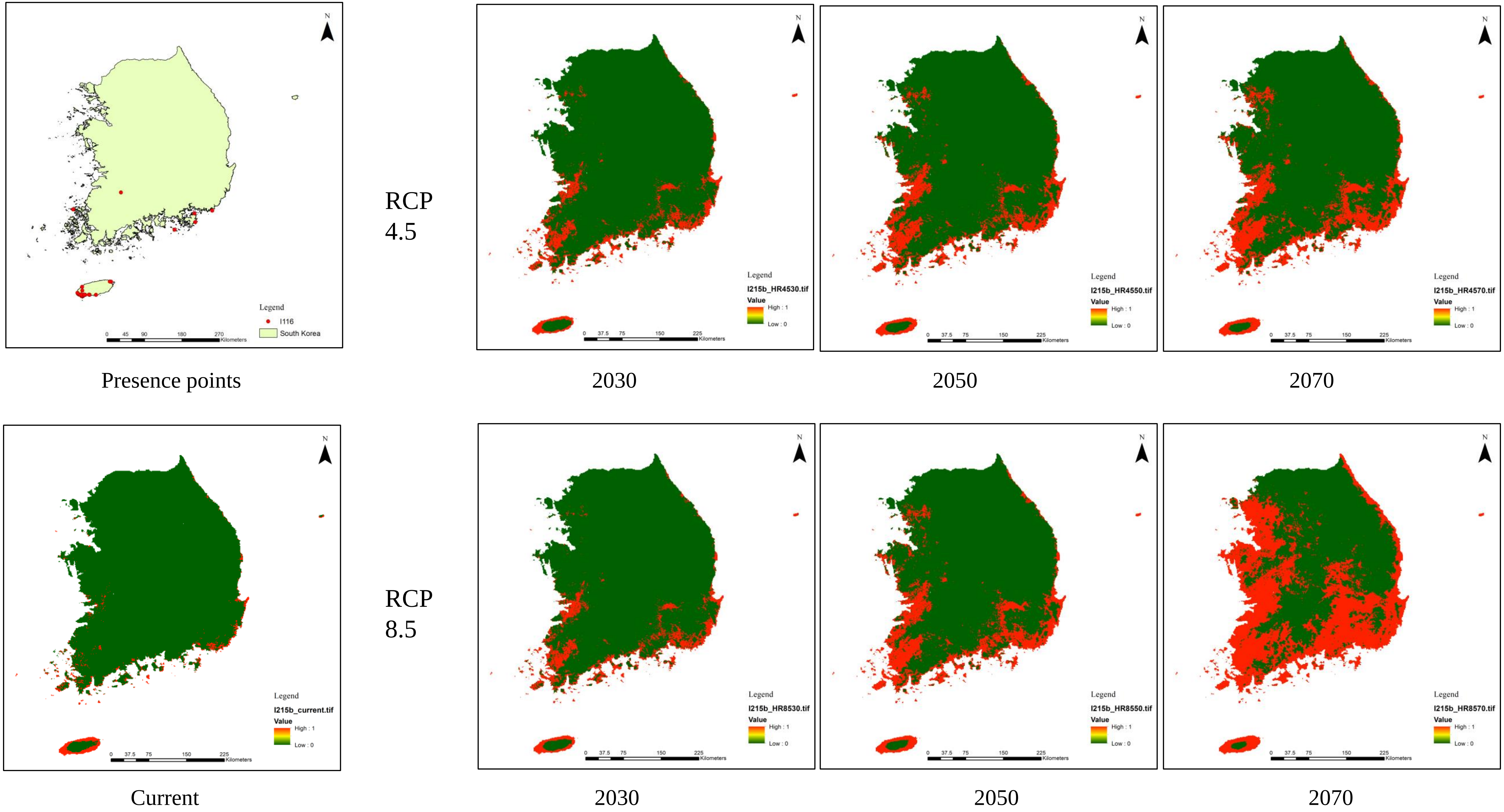

Figure S3p. Potential distribution of *Malva parviflora* (애기아욱) (I116) under RCP 4.5 and RCP 8.5.
