# Supplementary material for: Predicting Impacts of Climate Change on Northward Range Expansion of Invasive Weeds in South Korea
Source: Plants (Basel). 2021 Aug 5;10(8):1604. doi: 10.3390/plants10081604 (PMC8401637; doi:10.3390/plants10081604)
Supplement: Supplementary file 1 [file plants-10-01604-s001.zip › Supplementary Table S1.pdf]

Table S1. Pearson's correlation for bioclimatic variable selection

Variable	**Bio1**	Bio2	**Bio3**	**Bio4**	Bio5	Bio6	Bio7	**Bio8**	Bio9	Bio10	Bio11	**Bio12**	**Bio13**	**Bio14**	Bio15	Bio16	Bio17	Bio18	Bio19	**d-water**
Bio2	-0.28																			
**Bio3**	0.03	0.83																		
**Bio4**	-0.57	0.60	0.07																	
Bio5	0.85	0.20	0.31	-0.10																
Bio6	0.90	-0.58	-0.16	-0.84	0.56															
Bio7	-0.50	0.83	0.40	0.94	0.02	-0.82														
Bio8	0.92	-0.03	0.12	-0.24	0.96	0.69	-0.17													
Bio9	0.92	-0.46	-0.03	-0.80	0.62	0.97	-0.74	0.74												
Bio10	0.89	-0.01	0.08	-0.14	0.96	0.63	-0.10	0.98	0.68											
Bio11	0.95	-0.43	-0.01	-0.78	0.68	0.98	-0.72	0.79	0.98	0.73										
**Bio12**	0.04	-0.40	-0.24	-0.38	-0.22	0.23	-0.43	-0.14	0.26	-0.14	0.17									
**Bio13**	-0.48	0.19	-0.14	0.57	-0.30	-0.56	0.47	-0.32	-0.52	-0.25	-0.56	0.42								
**Bio14**	0.08	-0.44	-0.25	-0.43	-0.20	0.29	-0.49	-0.08	0.27	-0.13	0.23	0.41	-0.23							
Bio15	-0.48	0.44	-0.02	0.85	-0.12	-0.70	0.76	-0.21	-0.67	-0.12	-0.66	-0.19	0.77	-0.68						
Bio16	-0.32	-0.07	-0.23	0.23	-0.31	-0.29	0.13	-0.30	-0.25	-0.25	-0.32	0.74	0.88	-0.09	0.51					
Bio17	0.21	-0.51	-0.18	-0.67	-0.15	0.48	-0.68	-0.04	0.46	-0.09	0.41	0.51	-0.35	0.94	-0.83	-0.11				
Bio18	-0.37	0.11	-0.09	0.34	-0.27	-0.38	0.27	-0.28	-0.34	-0.24	-0.40	0.61	0.92	-0.16	0.60	0.96	-0.22			
Bio19	0.21	-0.51	-0.19	-0.67	-0.15	0.47	-0.68	-0.03	0.46	-0.09	0.41	0.52	-0.35	0.94	-0.83	-0.10	1.00	-0.22		
**d-water**	-0.13	-0.20	-0.15	-0.15	-0.27	0.01	-0.19	-0.22	0.02	-0.22	-0.04	0.21	0.00	0.27	-0.17	0.05	0.29	0.00	0.32	
**SSP1**	-0.09	0.00	0.02	-0.02	-0.11	-0.05	-0.01	-0.11	-0.05	-0.11	-0.06	0.02	0.02	-0.01	0.01	0.03	0.00	0.02	0.01	0.05
